# Biosensors of Wine Fermentation for Monitoring Chemical and Biochemical Interactions, Process Indicators and Migration of Compounds and Metabolites, Between Wine and Fermentation Vessels—A Critical Review

**DOI:** 10.3390/bios16030153

**Published:** 2026-03-10

**Authors:** Vasileios D. Prokopiou, Aikaterini Karampatea, Zoi S. Metaxa, Alexandros V. Tsoupras

**Affiliations:** 1 Hephaestus Laboratory, School of Chemistry, Faculty of Science, Democritus University of Thrace, Kavala University Campus, 65404 Kavala, Greece; vprokopi@chem.duth.gr (V.D.P.); zmetaxa@chem.duth.gr (Z.S.M.); 2 Department of Viticulture and Oenology, Democritus University of Thrace, 1st Km Dramas—Mikrochoriou, 66100 Drama, Greece; akarampa@vo.duth.gr

**Keywords:** wine fermentation, food contact materials, wine process safety, regulatory framework, HACCP monitoring, food safety

## Abstract

Wine alcoholic fermentation occurs in a dynamic biochemical environment where interactions between the vessel and the product can cause inorganic and organic species to migrate into the fermenting must or wine. At low pH and with rising ethanol levels, fermentation tanks made of stainless steel, concrete or cementitious materials, ceramics, or polymers exhibit material-specific behaviors that may promote the release of toxic trace elements or alter technologically important ions. These changes can affect yeast physiology, fermentation kinetics, and matrix stability, directly impacting wine safety and quality. They may also influence the evolution of key fermentation metabolites and phenolic constituents, thereby affecting process performance, color development, oxidative stability, and other quality-related attributes. This review synthesizes current evidence on migration mechanisms and examines how vessel composition shapes the chemical and microbiological profile of fermentation. It also critically evaluates biosensor technologies—covering both biorecognition elements and signal-transduction strategies—and assesses the transition from laboratory prototypes to in situ or at-line implementations capable of detecting both migration-related events and process-relevant compositional changes with operational value for HACCP-based control. Electrochemical, optical, bienzymatic, and nanozyme-enabled platforms are discussed in terms of selectivity, matrix compatibility, and long-term functional stability under polyphenol and protein interference, CO_2_ variability, fouling and biofouling, and calibration drift. Particular attention is given to analytes associated with vessel-derived migrants and to biosensor targets related to fermentation metabolites and phenolic indicators, which support dynamic process monitoring and quality-focused decision making. Considering regulatory compliance requirements across the EU, US, and Asia, we propose a practical pathway for integrating biosensors into HACCP monitoring by treating vessel–product interactions as critical control points, while laboratory reference methods remain essential for verification and compliance documentation.

## 1. Introduction

Alcoholic fermentation is the fundamental biotechnological process in winemaking and is recognized as a Critical Control Point (CCP) within HACCP systems. It involves intense microbial activity and rapid physicochemical changes that determine the safety and quality of the final product [1]. During fermentation, food safety risks may arise from endogenous compounds produced directly or indirectly by yeasts, such as biogenic amines and ethyl carbamate, as well as from exogenous contaminants, including heavy metals and other inorganic or organic migrants [2]. In this context, fermentation vessels are no longer viewed as passive containers but are redefined as active food contact materials (FCMs) that interact chemically with the fermenting must [3]. The aggressive environment of fermentation—characterized by low pH, the presence of ethanol, and thermal fluctuations—favors corrosion and material-dependent leaching, potentially leading to the migration of inorganic or organic constituents from the material matrix into the wine [4]. These vessel–matrix interactions may also influence the evolution of key fermentation metabolites and phenolic constituents, affecting chemical safety, fermentation kinetics, oxidative stability, color development, and overall product quality [5,6,7].

The economic and operational impact of these interactions is significant. The uncontrolled migration of substances not only poses toxicity risks to consumers but also disrupts the delicate chemical equilibrium of fermentation, affecting yeast metabolism and irreversibly altering the organoleptic profile of the product. Although the literature includes reviews on the development of biosensors for determining standard oenological parameters [8,9,10], a significant scientific and technological gap remains at the intersection of materials science and biosensing. In particular, limited attention has been given to biosensing strategies that link vessel-derived migration phenomena with process-level indicators, such as fermentation metabolites and phenolic parameters, which may provide complementary information on fermentation performance and wine quality evolution under real operating conditions [11,12]. Specifically, there is a lack of systematic investigation into the use of biosensors for monitoring the structural integrity of fermentation vessels and detecting the migration of constituents from tank walls to the must. Currently, control of such contaminants relies exclusively on conventional analytical methods [13,14]. While these methods are accurate, they are time-consuming, require expensive laboratory equipment, and provide only a static snapshot of quality, failing to capture the dynamic evolution of corrosion or leaching during fermentation. Consequently, there is no comprehensive approach evaluating how modern biosensors could replace cumbersome laboratory techniques and offer early warning solutions that are both economically viable and accessible to small and medium-sized enterprises (SMEs).

The aim of this work is to address this gap through a systematic analysis of fermentation vessels as active sources of contamination and process perturbation, and to evaluate the maturity of modern biosensor platforms for detecting the associated risks and compositional changes. The analysis goes beyond a simple inventory of sensors by considering vessel-derived substances not only as potential toxic contaminants for consumers, but also as factors capable of altering microbial physiology, fermentation kinetics, metabolite evolution, and phenolic profiles, ultimately affecting the organoleptic properties and technological stability of wine. Specifically, the objectives of this review are:

(a) Mapping migration mechanisms: To analyze the phenomena leading to the release of undesirable substances from the four main material categories under the specific conditions of alcoholic fermentation.
(b) Evaluating the analytical adequacy of biosensors: To examine available biosensing technologies for their ability to function reliably within the complex wine matrix in detecting migrating contaminants and monitoring process-relevant metabolites and phenolic indicators, with particular emphasis on selectivity against interferences, limits of detection (LODs), and the stability of biosensing materials.
(c) Proposing an integration framework: To develop a practical model for integrating sensors into production lines as tools for safety monitoring and process- and quality-oriented decision support, in a way that is economically viable for SMEs and supports the transition to digitalized winemaking.
(d) Highlighting regulatory and standardization gaps: To identify asymmetries in the current legislative framework regarding food contact materials and to propose measures for certifying the safety of biosensors themselves when immersed in food matrices.

## 2. Methods

This work is a structured narrative review with a transparent and reproducible search and source-selection process (PRISMA 2020-style flow diagram), aiming to critically synthesize evidence on (i) migration and leaching mechanisms of inorganic and organic species from wine fermentation vessel materials under real alcoholic fermentation conditions and (ii) biosensor technologies capable of supporting the detection of relevant migration events in must and wine matrices, with operational value for HACCP-oriented monitoring. The primary search window covered 2010–2025; earlier studies were included only when they introduced foundational concepts or pivotal proof-of-concept evidence directly relevant to the material–hydroalcoholic matrix interface.

Databases and search strategy. Literature was retrieved from Scopus and ScienceDirect, with PubMed additionally used to strengthen coverage of biosensor-related literature, particularly biorecognition and signal-transduction concepts. Searches used combinations of terms addressing: (a) wine and must fermentation and fermentation vessels as food-contact materials (stainless steel, ceramics/amphorae, concrete/cementitious materials, polymers), (b) corrosion, leaching, migration phenomena, and relevant analytes (migration, leaching, corrosion, trace metals, plasticizers, BPA, phthalates), (c) process-related compositional indicators, including ethanol, methanol, higher alcohols, acetaldehyde, organic acids, total phenolics, anthocyanins, and color- or oxidation-related phenolic parameters, and (d) biosensor technologies (aptamers, DNAzymes, molecularly imprinted polymers, electrochemical and optical sensing, screen-printed electrodes, anodic stripping voltammetry), explicitly considering matrix interference and surface contamination phenomena (fouling and biofouling) in complex matrices. A complementary identification strategy was applied through backward and forward citation chasing. The last search was performed on 6 March 2026.

Screening and eligibility criteria. Duplicate records were removed before title and abstract screening, followed by full-text assessment. Screening was performed by two authors, with disagreements resolved by consensus. Peer-reviewed original studies and reviews were included, along with selected authoritative regulatory or technical documents when necessary to substantiate food-contact compliance requirements and HACCP integration. Sources were excluded when they lacked substantive relevance to migration or leaching from food-contact materials under acidic or hydroalcoholic conditions, or to biosensors targeting relevant inorganic or organic analytes in complex matrices, or to biosensor platforms targeting fermentation metabolites and phenolic indicators relevant to process performance and wine quality monitoring, particularly when performance or validation evidence was insufficient or when interference and surface fouling or biofouling were not clearly addressed. Studies from other application domains (food and beverage, environmental, or biological samples) were included when they provided transferable evidence on design choices and operational robustness (interferences, fouling or biofouling, stability, calibration) critical for deployment in must and wine matrices.

**Figure 1:**
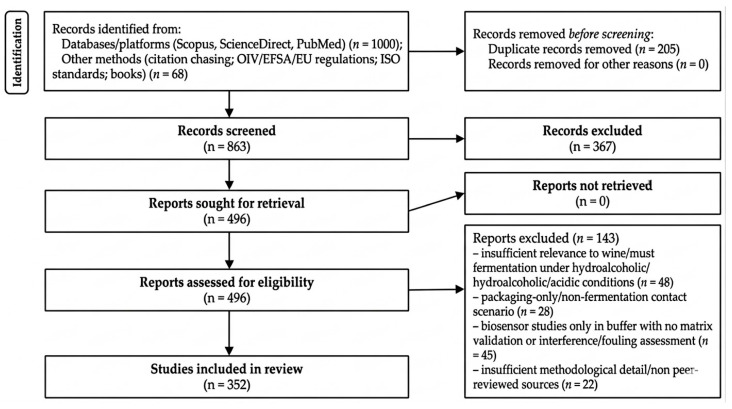
Flow diagram of the literature search, screening, eligibility assessment, and final selection of studies included in this narrative review.

Source quality assessment and synthesis. Sources were assessed using fit-for-purpose criteria to substantiate objectivity and scientific rigor. For migration and leaching studies, priority was given to clearly reported exposure conditions (pH, % ethanol, temperature, contact time), adequate material or surface characterization, and the use of established reference analytical methods. For biosensor studies, the assessment considered biorecognition and transduction type, limits of detection and linearity relative to critical thresholds, performance in real or equivalently challenging matrices (recovery, accuracy, repeatability), interference management, antifouling and anti-biofouling strategies, stability, and calibration requirements. For studies addressing fermentation metabolites and phenolic indicators, the assessment also considered their suitability for process monitoring, quality-oriented interpretation, and applicability under real or winery-relevant conditions. Meta-analysis was not performed because heterogeneity across studies (materials, exposure conditions, matrix types, and biosensor performance metrics) did not allow reliable statistical pooling; instead, a structured comparative synthesis was applied using summary tables and fit-for-purpose criteria to derive conclusions on technological readiness and field applicability. The identification, screening, and inclusion process is summarized in Figure 1.

## 3. Fermentation Contact Materials in Winemaking

### 3.1. Fermentation Vessels as Food Contact Materials

In modern winemaking, fermentation vessels are critical food contact materials (FCMs) because they remain in direct and prolonged contact with an acidic, ethanolic, and chemically active matrix [1,15]. Although often considered technologically neutral processing units, the physicochemical conditions of alcoholic fermentation (pH 3–4, presence of ethanol, sulfur dioxide, organic acids, and fluctuating temperature) can promote corrosion, leaching, or material aging, leading to the migration of inorganic or organic constituents into the fermenting medium [16,17,18].

Stainless steel remains the dominant material for fermentation tanks due to its high corrosion resistance, non-porous and hygienic surface, and capacity for precise temperature control. The most commonly used grades are AISI 304 and AISI 316, the latter offering enhanced resistance to acidic and chloride-rich environments due to the presence of molybdenum [19,20]. Nevertheless, the acidic nature of grape must and wine, combined with ethanol, SO_2_, and organic acids, may weaken the passive oxide layer and result in the release of Ni^2+^, Cr^3+^, and Fe^2+^/Fe^3+^ ions into the fermenting matrix [21]. Although most available studies originate from other acidic food systems or simulants, increased Ni and Cr concentrations have been reported under low-pH conditions [17]. The extent of metal release depends on alloy quality, surface integrity, must composition, contact time, temperature fluctuations, and cleaning-in-place (CIP) procedures. Nickel is of particular concern due to its allergenic potential and, together with chromium, is among the elements monitored under European and international food contact material regulations [22]. Consequently, stainless steel cannot be considered fully inert under fermentation conditions [23,24].

Ceramic vessels, including traditional and modern amphorae, have re-emerged in winemaking but present distinct challenges from a food contact material perspective [25,26]. The mineralogical composition of the clay and, most importantly, the quality, composition, and stability of the glaze determine the risk of heavy metal migration. The release of Pb^2+^ and Cd^2+^ into acidic and ethanolic matrices is well documented, particularly when glazes are of poor quality, aged, or insufficiently fired [27,28,29]. Experimental data using model wine solutions (12% ethanol, pH ~3.5) confirm measurable leaching of Pb and Cd from degraded or unevenly glazed surfaces [28,30]. Although model systems do not fully reproduce the dynamic conditions of fermentation—such as CO_2_ evolution, temperature fluctuations, and the presence of colloidal components—they indicate that surface stability is a critical safety factor. In the European Union, ceramic food contact materials are regulated through specific migration limits for Pb and Cd (Directive 84/500/EEC and its amendment 2005/31/EC) [31,32]. However, compliance studies show that traditional or artisanal vessels may exceed these limits [33]. Mitigation strategies, including low-lead or lead-free glazes, high-temperature firing, and internal coatings, may introduce additional sources of organic migration under acidic and ethanolic conditions [34].

Concrete and cement-based tanks are an emerging trend, particularly in small-scale and quality-oriented winemaking [35]. Despite their technological advantages, their chemical inertness under fermentation conditions remains poorly characterized [36]. Available evidence is largely derived from drinking water studies, where the release of Ca^2+^, K^+^, Al^3+^, and trace metals has been reported as a result of hydration and carbonation processes within the cementitious matrix [37]. The dissolution of Ca(OH)_2_ followed by CaCO_3_ formation may increase porosity and promote element release [38,39]. Although fermentation conditions differ substantially—due to acidic pH, ethanol, and phenolic compounds—these findings suggest that concrete is not inherently inert. The potential release of Ca^2+^ may affect wine ionic balance and contribute to tartrate precipitation, with implications for product stability. From a regulatory standpoint, cement-based materials are not covered by harmonized EU-specific migration limits beyond the general safety requirement of Regulation (EC) No 1935/2004 [3], creating uncertainty regarding long-term safety assessment.

Polymeric vessels (PET, HDPE, PP) are primarily used as low-cost solutions but present significant challenges as food contact materials [40]. The acidic and ethanolic nature of grape must can promote the migration of residual catalysts or additives. PET has been associated with antimony (Sb) release, especially at higher ethanol concentrations or temperatures [41]. In HDPE and PP, the release of monomers, oligomers, and low-molecular-weight compounds has been reported, particularly after repeated use [42]. Additionally, plasticizers such as phthalates (DEHP, DBP) and bisphenol A (BPA) have been detected in wines and other beverages at μg/L levels [43]. These substances are regulated by specific migration limits under Regulation (EU) 10/2011 [44], but compliance is strongly affected by polymer type, contact duration, acidity, ethanol concentration, and temperature [45,46]. Occasional use of non-certified containers in small wineries further increases potential risk.

**Figure 2:**
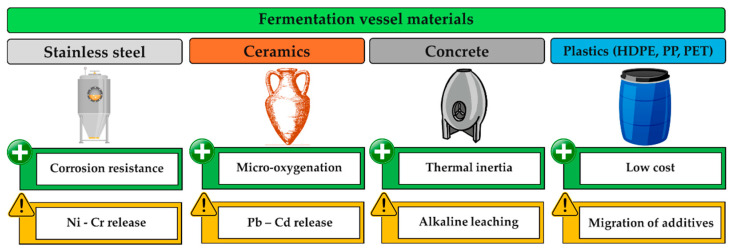
Fermentation vessel materials and their migration-related risks.

Overall, analysis of the main categories of fermentation vessels shows that no material is completely inert under winemaking conditions. Migration of inorganic and organic constituents depends on material type, surface condition, and the dynamic physicochemical environment of fermentation. The lack of systematic studies under real fermentation conditions—particularly for non-conventional materials such as concrete—underscores the need for targeted monitoring strategies. Figure 2 schematically summarizes the main categories of fermentation vessels, highlighting their key functional advantages and the specific migration-related risks associated with each material.

### 3.2. Migration Mechanisms Under Alcoholic Fermentation Conditions

The migration of chemical constituents from fermentation vessels into grape must or wine is governed by a combination of physicochemical factors that change dynamically throughout alcoholic fermentation [47,48]. These conditions differ substantially from those used in standard migration tests or simplified food simulants, making material behavior highly dependent on the actual processing environment [49]. The low pH of grape must and wine (typically 3–4), along with the presence of organic acids (tartaric, malic, lactic), increases the solubilization of inorganic components and promotes corrosion or leaching at material surfaces. Acidic conditions can destabilize passive metal layers, enhance the dissolution of metal oxides, and accelerate the migration of heavy metals or alkali and alkaline earth ions [49,50,51]. Ethanol, whose concentration progressively increases during fermentation, acts as a co-solvent and enhances the solubility of hydrophobic or partially polar organic compounds. This effect is particularly relevant for the migration of organic additives, monomers, or plasticizers from polymeric materials or surface coatings [52]. The combined action of ethanol and acidic pH substantially alters migration mechanisms compared with non-alcoholic food matrices [53].

Sulfur dioxide (SO_2_), present in both free and bound forms, affects the redox environment of fermentation and may contribute to chemical changes at material surfaces, particularly metallic ones [54]. Fluctuations in redox potential during fermentation can influence the stability of metal ions and the chemical forms in which they migrate into the liquid matrix [55]. Contact time is a critical parameter, as fermentation involves prolonged interaction between the material and the product, often extending over several weeks [56]. Temperature variations, whether controlled or localized, can accelerate the kinetics of diffusion and chemical reactions, thereby enhancing migration, particularly in porous or coated materials [57]. Cleaning and sanitization practices (cleaning-in-place, CIP) have a decisive impact on the surface condition of fermentation vessels. Acidic or alkaline detergents, oxidizing agents, and mechanical stress can alter protective surface layers, increase roughness, and accelerate material aging. These effects are cumulative and become evident after repeated use cycles, complicating the prediction of long-term material behavior [58,59].

A large proportion of the available literature relies on model solutions or food simulants that do not fully reproduce the dynamic nature of alcoholic fermentation, including ethanol evolution, CO_2_ production, compositional changes, and the presence of colloidal and microbial components. Conclusions drawn from such systems must therefore explicitly acknowledge their limitations, as migration phenomena under real fermentation conditions may differ both qualitatively and quantitatively.

### 3.3. Impact of Vessel-Derived Metals on Key Fermentation Metabolites and Phenolic Compounds

The migration of metals and ions from food contact materials during alcoholic fermentation is not only a compliance issue; it can also cause measurable changes in the metabolic fingerprint of must or wine, because ions act (i) as enzyme cofactors and regulators of yeast homeostasis (Mg, K, Ca) and (ii) as catalysts of redox and oxidative reactions (mainly Cu and Fe), which affect the alcohol–aldehyde–acid balance. Fermentation has a distinctive dynamic: essential ions tend to be retained or accumulated, while transition and heavy metals often decrease in the liquid phase due to binding to lees, without negating their biochemical impact during the critical period of active fermentation [60,61].

For alcohols, the immediate impact of ion migration and availability is reflected mainly in ethanol yield and the profile of higher alcohols (fusel alcohols). Adequate Mg and K support yeast physiology and favor higher production of ethanol and aroma-related by-products, whereas excess Ca may inhibit fermentation by competing with Mg uptake, leading to lower fermentation performance [62,63,64]. Conversely, when transition or toxic metals migrate or are present at elevated levels, stress and inhibition are observed and performance tends to deteriorate; a characteristic example is Cu, where copper stress alters the growth and fermentative properties of *S. cerevisiae* and may reduce ethanol production at higher concentrations [65].

For acetaldehyde, the migration or presence of Cu and Fe has a particularly direct effect, because these metals favor oxidative pathways and redox cycling that shift the balance toward carbonyl formation. It has been shown that Cu(II) levels in must affect acetaldehyde concentration and simultaneously modify phenolic composition and color characteristics in red and white wines [66]. Similarly, Fe in an acidic–alcoholic environment is linked to the cycle of oxidative species [67], explaining why changes in metal speciation during fermentation may increase the acetaldehyde burden when suitable oxidative conditions are present.

For organic acids, the most visible immediate indicator is volatile acidity (acetic acid), which responds strongly to ionic balance and yeast metabolic stress. Available evidence shows that Mg and K are generally associated with more favorable fermentation and reduced acetic acid production, whereas high Ca tends to hinder fermentation progress (Mg–Ca antagonism) and lead to increased acetic acid. These observations and mechanistic interpretations are supported by studies on the role of inorganic elements in fermentation and by-products [63,64] and by experimental data showing that the addition of metal ions affects the evolution and removal of acetic acid during fermentation [68]. In parallel, transition metals (Cu and Fe) can increase the oxidative pressure of the system and shift equilibria toward more oxidized products, thereby affecting the overall profile of organic acids [61,65]. For heavy metals such as Pb and Cd, the direct product impact is mediated through yeast toxicity and disruption of essential metal homeostasis. This can manifest as sluggish or inhibited fermentation with a consequent shift in the metabolic profile (reduced ethanol and higher alcohols, and altered organic acids), even if part of these metals is removed from the liquid phase due to binding to lees. Relevant biological evidence is documented by studies on the effects of Cd on *S. cerevisiae* metabolism and essential metals [69] and by a genomic approach to Pb sensitivity [70].

The migration of metal ions from fermentation vessels into must or wine can influence the chemical behavior of phenolic compounds, primarily through complexation, redox processes, and induced polymerization. Redox-active metals such as Fe^2+^/Fe^3+^ and Cu^2+^, in particular, accelerate the oxidation of phenolics to quinones, increase oxygen consumption, and contribute to changes in wine color and astringency [71,72,73]. These interactions are especially important for anthocyanins and tannins, as they may enhance or destabilize copigmentation phenomena and affect color stability [74]. In contrast, Ca^2+^, Mg^2+^, and K^+^ do not exhibit strong redox activity but modify the ionic balance and the stability of complexes or precipitates, indirectly influencing wine structure and mouthfeel [75,76]. Al^3+^ can also form complexes with flavonoids and anthocyanin structures, affecting the chemical and color stability of phenolic systems [77]. Additionally, Pb^2+^, Cd^2+^, and Ni^2+^ bind to phenolic or macromolecular fractions of wine, influencing their chemical form and bioavailability, while the behavior of chromium depends on its speciation, with Cr(III) showing a greater tendency for complexation and Cr(VI) exhibiting a stronger oxidative character [78,79,80]. Overall, pH, phenolic structure, and the presence of metal ions determine the stability of these complexes and ultimately influence the evolution of the sensory characteristics of wine [81,82].

### 3.4. Critical Control Points (CCPs) During Alcoholic Fermentation Related to Vessel–Product Interactions

Alcoholic fermentation is a highly complex processing stage in which continuously changing physicochemical and biological conditions can make the interaction between the fermentation vessel and the product a critical food safety factor [83]. According to Hazard Analysis and Critical Control Point (HACCP) principles, these interactions can be identified as Critical Control Points (CCPs), particularly when materials that are not fully inert remain in prolonged contact with the fermenting matrix [84]. Fermentation vessels are a primary CCP because they can release inorganic or organic substances under acidic and ethanolic conditions [85]. The release of metal ions from stainless steel surfaces when the passive layer is compromised, the migration of Pb and Cd from ceramic glazes, the leaching of ions from cementitious matrices, and the release of organic additives from polymeric materials are documented or potential chemical hazards directly associated with the fermentation stage [48,86,87,88,89]. The nature and extent of these risks depend on vessel composition, surface condition, contact time, and processing conditions.

In addition to direct chemical migration, fermentation vessels may indirectly affect food safety by influencing fermentation kinetics. Parameters such as oxygen transfer, thermal inertia, and CO_2_ release impact yeast metabolism and microbial dynamics, potentially creating secondary CCPs related to sluggish or stuck fermentations and the subsequent formation of undesirable metabolites, including biogenic amines, volatile sulfur compounds, and increased volatile acidity [90,91]. Although these compounds do not originate from the vessel material itself, vessel–process interactions can enable their formation. From a food safety perspective, particular concern arises from chemical hazards with low acceptable limits and cumulative toxicological effects, such as heavy metals and industrial organic contaminants [92,93]. The literature identifies significant knowledge gaps regarding the behavior of non-conventional fermentation vessels, especially concrete tanks, under real winemaking conditions [88,89]. The lack of quantitative migration data limits the definition of precise critical limits and constrains the effective implementation of HACCP-based control strategies.

Beyond the formation of undesirable safety-related metabolites, vessel–product interactions may also be evident in measurable changes in key fermentation metabolites and phenolic parameters. Variations in ethanol production, higher alcohol formation, acetaldehyde accumulation, organic acid balance, and phenolic evolution do not alone define vessel-related CCPs, but they may serve as early indicators of process disturbances caused by ionic imbalance, oxidative stress, or vessel-derived migration phenomena. In this context, such compositional changes are highly relevant within HACCP-based control, as they provide supporting evidence that the interaction between the fermentation vessel and the product is beginning to affect both process performance and product quality under real winemaking conditions.

Therefore, fermentation vessels should not be viewed solely as technological equipment but as active process elements that can influence both product safety and stability. Recognizing vessel–product interactions as CCPs underscores the need for systematic monitoring of critical parameters during fermentation and for developing evidence-based risk management strategies tailored to real winemaking conditions. This monitoring framework should address not only direct migration hazards but also selected process and quality indicators, including fermentation metabolites and phenolic parameters, when these provide early evidence of vessel-related process deviation under actual fermentation conditions. Accordingly, vessel–product interactions should be considered material-specific Critical Control Points (CCPs), as they may affect safety and process stability through direct migration phenomena and related quality deviations, as summarized in Table 1.

## 4. Technological Landscape of Biosensors for Chemical Contaminants in Wine Fermentation

This section outlines the current technological landscape of biosensors developed for detecting chemical contaminants during wine fermentation. The analysis covers both metallic and organic analytes, including heavy metals, cations, plasticizers, and phenolic compounds, with emphasis on the biorecognition elements used, the signal transduction strategies implemented, and the reported analytical performance characteristics, as examined in the previous section.

### 4.1. Biorecognition Platforms for Heavy Metals

Having established the risk of toxic heavy metal migration from fermentation vessel materials into the must, the next critical challenge is their reliable and selective detection. Unlike the quantification of conventional oenological parameters, determining trace-level heavy metals in a chemically complex, acidic, and ethanol-rich matrix requires advanced bioanalytical platforms capable of functioning under significant matrix interference.

#### 4.1.1. Biorecognition Elements

In contrast to macroelements that regulate the physicochemical stability of wine, detecting heavy metals such as lead (Pb^2+^), cadmium (Cd^2+^), nickel (Ni^2+^), chromium (Cr(III)/Cr(VI)), and aluminum (Al^3+^) presents distinct analytical challenges. These require strategies capable of identifying trace-level concentrations (ppb) within a chemically saturated environment [60,101]. The fundamental difficulty arises from the limited availability of highly selective natural receptors suitable for analytical applications, as most of these metals do not fulfill essential biological roles comparable to iron. Consequently, research has increasingly focused on developing synthetic and biomimetic recognition systems beyond conventional enzyme inhibition approaches [102,103,104].

For Pb^2+^ and Cd^2+^, earlier analytical methods relied on enzyme inhibition, including urease and oxidases. Although cost-effective, these systems exhibit limited target discrimination, particularly under the acidic and ion-rich conditions of wine, where competing ions and low pH may induce false-positive responses [105,106]. DNAzymes and aptamers have therefore emerged as more robust alternatives. Catalytic DNAzymes such as GR-5 exploit Pb^2+^-induced site-specific DNA cleavage, enabling metal-specific signal generation under optimized ionic conditions [107,108,109,110]. For Cd^2+^, several aptamers adopt G-quadruplex conformations, while others rely on stem–loop structures [111,112]. Compared to protein-based receptors, nucleic acid platforms demonstrate improved operational stability in ethanol-containing environments, supporting sensor regeneration and reuse [113].

Detection of Ni^2+^ and chromium species presents additional complexity due to the scarcity of intrinsically selective biological receptors. While chemical complexing agents such as dimethylglyoxime (DMG) remain widely used for Ni^2+^ determination, biosensor development increasingly explores modified peptides (e.g., L-cysteine) and aptamers [114,115]. Molecularly imprinted polymers (MIPs) offer a promising alternative, providing synthetic recognition cavities tailored to Ni^2+^ and enhanced tolerance under acidic conditions [116]. For chromium, analytical relevance lies in distinguishing Cr(III) from the more toxic Cr(VI). Although whole-cell biosensors have been proposed for assessing bioavailability, functional nucleic acids and MIPs are generally favored for rapid and matrix-resilient detection of specific chromium species [117,118].

Al^3+^ represents a distinct case, as it lacks a defined physiological metabolic function. Detection strategies often draw inspiration from microbial iron-acquisition mechanisms, employing siderophores such as alcaligin and enterobactin. Despite their primary affinity for Fe^3+^, these ligands also exhibit strong binding affinity toward Al^3+^ [119]. To improve analytical discrimination and mitigate competitive binding from other metal ions, such systems are often integrated with fluorescence-based reporting mechanisms activated upon target coordination [120].

Overall, the literature indicates a progressive shift toward functional nucleic acids and biomimetic polymers for heavy metal sensing. While enzyme inhibition remains relevant for general toxicity screening, it does not provide the metal-specific resolution required for compliance with stringent maximum residue limits (MRLs) established for individual metal ions such as Pb^2+^ and Cd^2+^. Aptamers and DNAzymes, offering nanomolar to picomolar detection ranges along with enhanced chemical robustness, are increasingly adopted as reference recognition platforms for safety-oriented biosensing in fermentation systems. The main biorecognition elements currently used for the selective detection and speciation of heavy metal ions are summarized in Table 2.

#### 4.1.2. Signal Transduction Mechanisms

While the bioreceptor forms the recognition core of a biosensing system, the transducer provides the amplification mechanism that converts molecular interactions into a measurable analytical signal reflecting the actual concentration of heavy metals. In detecting toxic metals such as Pb^2+^, Cd^2+^, Ni^2+^, Cr(III)/Cr(VI), and Al^3+^, the primary challenge for the transduction platform lies not only in translating a chemical event into an electrical or optical output but also in achieving a signal-to-noise ratio (S/N) compatible with stringent regulatory maximum residue limits (MRLs) [101,129]. The literature consistently highlights a methodological distinction between electrochemical techniques, which benefit from intrinsic preconcentration capabilities, and optical approaches, which exploit nanophotonic enhancement mechanisms [113,130].

For electroactive metals such as Pb^2+^ and Cd^2+^, anodic stripping voltammetry (ASV) remains one of the most established and sensitive electrochemical techniques for trace metal detection [131,132]. Its analytical advantage over conventional potentiometric methods derives from the electrolytic preconcentration step, during which the metal ion accumulates on the electrode surface prior to measurement, enabling detection at sub-μg/L concentrations [133]. However, application in wine matrices requires mitigation of electrode fouling caused by polyphenolic compounds [134,135]. A widely adopted strategy involves using screen-printed electrodes (SPEs) modified with nanomaterials such as bismuth nanoparticles or carbon nanotubes. As disposable, low-cost platforms, SPEs reduce memory effects and minimize cleaning requirements, while their nanostructured surfaces increase the effective electroactive area [136,137,138,139]. For chromium, electrochemical approaches can enable selective detection of Cr(VI) and, under appropriately optimized protocols, differentiation between Cr(III) and Cr(VI), although careful method design and calibration remain essential [118,127,140]. Metals exhibiting limited or less favorable electrochemical responses under mild aqueous conditions, such as Al^3+^ and, in certain configurations, Ni^2+^, are more frequently addressed through optical platforms. In these systems, aptamers or DNAzymes are coupled with fluorescence-based reporters, including fluorescence resonance energy transfer (FRET) architectures, allowing conformational changes upon target binding to be monitored optically [109,110,113,141,142].

A significant analytical limitation arises from the intense coloration of red wines, which can absorb excitation or emission wavelengths of conventional fluorophores, leading to inner filter effects. To mitigate this interference, upconversion nanoparticles (UCNPs) or quantum dots operating in the near-infrared (NIR) spectral region have been employed as alternative signal reporters. Within this spectral window, wine matrices exhibit increased optical transparency, facilitating sensitive detection of Al^3+^ and Ni^2+^ with reduced matrix interference and, in some cases, minimal sample pretreatment [143,144,145].

Quartz crystal microbalance (QCM) sensors represent a less widely implemented but analytically valuable label-free alternative. Detection is based on frequency shifts resulting from mass changes upon metal binding at the sensor surface [135,146]. Although highly sensitive, QCM measurements in viscous matrices such as sweet wines require strict control of temperature and rheological parameters, which may limit their routine use outside controlled laboratory settings [147,148].

Overall, the selection of a transduction strategy for heavy metal monitoring is governed by both the physicochemical characteristics of the target ion and the complexity of the wine matrix. Electrochemical approaches, particularly ASV, remain highly suitable for redox-active metals such as Pb^2+^, Cd^2+^, and Zn^2+^ due to their favorable sensitivity-to-cost ratio. In contrast, optical transduction enhanced by advanced nanomaterials provides an effective pathway for metals with limited electrochemical activity, including Al^3+^ and, under specific configurations, Ni^2+^. Accordingly, Table 3 summarizes the main transduction mechanisms for heavy metal sensing, along with their advantages, limitations, and suitability in wine matrices.

#### 4.1.3. Analytical Validation and Critical Performance Characteristics

The successful transition of biosensors from laboratory research to industrial food safety control requires rigorous validation of their analytical reliability against established reference methods, such as inductively coupled plasma mass spectrometry (ICP-MS) and graphite furnace atomic absorption spectrometry (GFAAS). Given the extremely low regulatory limits (MRLs) for heavy metals in wine (e.g., Pb^2+^ < 150 μg/L; Cd^2+^ < 10 μg/L), performance evaluation must balance high analytical sensitivity with robustness against matrix interference [152,153].

In terms of sensitivity and limits of detection (LOD), advances in nanobiotechnology have significantly narrowed the performance gap between biosensors and conventional spectroscopic techniques [23,24,104]. Electrochemical biosensors based on anodic stripping voltammetry (ASV), using bismuth-modified or carbon nanotube-modified electrodes, report detection limits for Pb^2+^ and Cd^2+^ in the range of 0.1–1.0 μg/L, especially after appropriate dilution or minimal sample pretreatment [131,132,136,137,149]. These values are comparable to those achieved by GFAAS and meet the regulatory requirements established by the OIV, supporting their suitability for compliance testing [153,154]. Optical platforms based on aptamers and DNAzymes enhanced with nanomaterials (e.g., FRET architectures or UCNPs) also demonstrate picomolar-level sensitivity [109,110,141]. Although ICP-MS retains superior absolute sensitivity at the ppt level, such performance often exceeds routine monitoring requirements, as biosensor detection limits are already sufficient for regulatory compliance [13,155].

Biosensors offer a distinct advantage in metal speciation. For chromium, conventional ICP-MS quantifies total chromium content and requires coupling with high-performance liquid chromatography (HPLC) for speciation [156,157]. In contrast, biosensors employing selective DNAzymes or molecularly imprinted polymers (MIPs) can be designed to respond preferentially to Cr(VI), providing targeted information on the more toxic species [118,127,128,140]. Additionally, biosensors often measure the free or bioavailable fraction of metal ions, while reference methods typically involve complete sample digestion [21]. While this may yield results that differ from total metal quantification, it provides complementary insight into the potentially bioactive fraction relevant to consumer exposure and fermentation processes.

Matrix effects remain a central challenge in analytical validation due to the chemical complexity of wine [101]. Spiking studies in authentic wine samples report recovery values between 85% and 115% for electrochemical sensors, with relative standard deviations (RSD) typically below 10% across replicate measurements—values consistent with AOAC performance criteria [134,158]. Interference management varies by analyte: for Al^3+^, optical measurements may require controlled dilution (1:10 to 1:50) or near-infrared fluorescence strategies to mitigate inner filter effects, while electrochemical detection of Pb^2+^ and Cd^2+^ generally shows greater matrix tolerance [120,135,143,144]. Incorporating Nafion membranes to exclude surface-active interferents has further reduced fouling-related errors, enabling relative deviations below 5% compared to ICP-MS measurements under optimized conditions [159].

Beyond analytical accuracy, operational viability is supported by sensor stability and reusability. Aptamer- and DNAzyme-based systems can often be regenerated with chelating solutions (e.g., EDTA), with several studies demonstrating more than 50 reuse cycles and signal loss below 10%. Replacing enzyme-based recognition elements with nucleic acids has also improved shelf life, allowing storage for extended periods at ambient temperature. Overall, analytical validation indicates that heavy metal biosensors are evolving from purely experimental tools to reliable complementary technologies that, despite a modest difference in absolute accuracy compared to ICP-MS, provide added functional value through speciation capability and field-deployable operation [103,104]. Table 4 highlights the main analytical and operational differences between heavy metal biosensors and conventional reference methods.

### 4.2. Biorecognition Platforms for Ions

Monitoring metal ions during alcoholic fermentation is not only an analytical requirement but also a critical tool for controlling the technological and organoleptic development of wine. Cations in the fermentation matrix affect physicochemical stability, biochemical kinetics, and compliance with regulatory safety limits, while their concentrations change dynamically due to acidity, ethanol production, and yeast metabolic activity. The need for reliable, rapid, and in situ analysis in such a complex environment requires systematic evaluation of modern biorecognition strategies, signal transduction mechanisms, and key performance parameters that determine biosensor functionality. In this context, this subsection critically examines current technological approaches, emphasizing their compatibility with the demanding physicochemical conditions of active wine fermentation [5,162].

#### 4.2.1. Biorecognition Elements

The effectiveness of biosensor platforms for monitoring fermentation products depends primarily on the choice of biorecognition element, which must maintain thermodynamic stability and high selectivity in a chemically demanding environment. The wine matrix is characterized by low pH (3.0–4.0), the presence of ethanol, and a high concentration of phenolic compounds—conditions under which conventional enzymatic systems often show performance limitations. Although enzymes offer intrinsic specificity, their tertiary structure is prone to partial denaturation under these conditions, which can reduce catalytic activity and compromise measurement reliability. As a result, research has increasingly focused on biomimetic and synthetic approaches that provide greater structural robustness and analytical stability [5,134,163].

For macroelements that regulate physicochemical stability, such as calcium (Ca^2+^), magnesium (Mg^2+^), and potassium (K^+^), Molecularly Imprinted Polymers (MIPs) have emerged as robust and reliable recognition platforms. Unlike enzymatic systems, MIPs act as synthetic receptors with predefined recognition cavities, offering increased resistance to acidification and ethanol-induced solvation [164,165]. Their structural stability makes them particularly suitable for applications such as tartrate precipitation monitoring [166,167]. In contrast, classical ionophores used in potentiometric configurations, while attractive for their rapid response, may experience cross-sensitivity among ions with similar charge and ionic radius. This limitation can be addressed by integrating them into electronic tongue arrays, where multivariate chemometric algorithms enable effective discrimination of overlapping signals [168,169,170,171].

Transition metals involved in oxidative spoilage phenomena (casse), such as iron (Fe^2+^/Fe^3+^) and copper (Cu^2+^), require highly selective recognition due to their low concentrations and dynamic redox equilibria [5]. Microbial siderophores, such as pyoverdine, show high binding affinity for iron; however, selectivity may be reduced in complex matrices due to competing cations. For copper detection, chemical functionalization of sensor surfaces with heterocyclic ligands (e.g., quinoline, pyridine) enables stable chelate complex formation even under acidic conditions, achieving nanomolar detection limits and significantly reducing interference effects [5,172,173,174,175].

Zinc (Zn^2+^) detection increasingly favors aptamer-based systems over immunochemical methods. Antibodies tend to undergo structural destabilization in hydroalcoholic environments, whereas aptamers (DNA/RNA oligonucleotides) exhibit greater conformational resilience and reversible folding for target binding. Their regenerative capability and operational stability make them suitable for continuous monitoring during fermentation [163,176]. Overall, the shift from fragile enzymatic systems to synthetic receptors and aptamer-based architectures represents a strategic direction for developing biosensors compatible with modern oenological requirements. This trend is also shown in Table 5, which presents the performance and suitability of various biorecognition elements for ion sensing in complex winemaking environments.

#### 4.2.2. Signal Transduction Mechanisms

The operational performance of biosensors during alcoholic fermentation depends largely on the ability of the transduction system to maintain a stable and reproducible signal in a chemically demanding environment. Unlike trace-level toxic metal analysis, where achieving extremely low detection limits is the primary goal, monitoring major cations during fermentation prioritizes baseline stability, resistance to surface fouling, and continuous real-time functionality. The fermentation matrix is characterized by high ionic strength, low pH, increasing ethanol concentration, and the presence of proteins and polyphenolic compounds. These factors can interfere with charge transfer processes in electrochemical systems and affect light propagation or absorption in optical configurations. Therefore, the selection and engineering of the transducer are critical design parameters for reliable in situ monitoring [5,134,162].

Electrochemical approaches, particularly potentiometric and conductometric systems, are widely used for monitoring ions such as Ca^2+^, Mg^2+^, and K^+^. Their appeal lies in minimal sample preparation and rapid response times. However, in fermentation environments, adsorption of organic constituents onto electrode surfaces can cause baseline drift and reduced reproducibility. Surface modification strategies using nanostructured materials or selective membranes aim to improve conductivity and mitigate fouling effects, thereby enhancing long-term operational stability [5,134,177,178,179,180]. Optical techniques are typically chosen when enhanced selectivity is required or when electrochemical responses are limited under mild aqueous conditions. However, chromophoric compounds present in wine, such as anthocyanins and tannins, may compromise analytical accuracy through absorption phenomena and inner filter effects. To address these limitations, detection systems operating in spectral regions with reduced matrix interference are preferred, along with nanomaterials exhibiting improved photophysical stability [5,134,181,182,183].

Mass-sensitive transduction mechanisms, such as quartz crystal microbalance (QCM) sensors, offer a label-free alternative based on frequency shifts induced by mass changes upon ion binding. While highly sensitive, their use in viscous or rheologically variable fermentation systems requires strict control of temperature and hydrodynamic conditions, which may limit their routine industrial application. Overall, selecting a transduction strategy for fermentation monitoring involves balancing analytical sensitivity with operational robustness. Electrochemical methods offer practicality and suitability for continuous monitoring, while optical and mass-sensitive approaches provide complementary solutions when enhanced selectivity or alternative detection principles are needed [5,134,135,162]. These considerations are reflected in Table 6, which compares the main signal transduction mechanisms used for ion sensing in fermentation monitoring.

#### 4.2.3. Analytical Validation and Critical Performance Characteristics

The transition of biosensors from the laboratory to real industrial applications requires rigorous validation of their analytical performance against established reference methods, such as ICP-MS and AAS. This evaluation goes beyond a numerical comparison of detection limits and examines the functional behavior of the systems within the complex physicochemical environment of fermentation products, with emphasis on selectivity, sensitivity, response time, and long-term stability [5,134,162].

Selectivity is a particularly critical parameter, as the wine matrix is a chemically complex environment rich in organic acids, polyphenols, and ethanol. While ICP-MS and AAS significantly reduce organic interferences through thermal decomposition or ionization, biosensors operate under mild conditions, making them susceptible to nonspecific adsorption and cross-sensitivity effects [5,134,180]. The literature indicates that using individual sensors may lead to deviations due to the presence of ions with similar characteristics (e.g., Ca^2+^ and Mg^2+^). However, the integration of chemometric tools and the development of sensor arrays (“electronic tongues”) have, in some cases, enabled performance approaching that of reference methods without the need for extensive sample pretreatment [168,170,171,186,187]. Regarding sensitivity and limit of detection (LOD), modern biosensors achieve nanomolar (nM) levels for metals such as copper and zinc, as well as concentrations in the mg/L range for macroelements such as calcium [172,173,174,179]. In absolute terms, ICP-MS provides lower detection limits, often in the ppt range [153]. Nevertheless, its extremely high sensitivity frequently exceeds the analytical requirements of routine control in the food industry, where regulatory limits and concentrations affecting physicochemical stability are at significantly higher levels. In this context, biosensors may be considered “fit for purpose,” offering sufficient accuracy with lower operational cost and simplified infrastructure [5,134,162].

A significant comparative advantage of biosensors is response time. Laboratory analyses using AAS or ICP-MS require sample transport and preparation procedures such as digestion, dilution, and calibration, while biosensors can provide results in real time or within a few minutes. This enables continuous monitoring of fermentation kinetics and the implementation of immediate corrective actions, a capability not practically achievable with conventional laboratory techniques [5,134,162]. A primary limiting factor of the technology remains the stability and lifetime of the biorecognition elements. The acidic environment (pH 3.0–4.0) and the presence of ethanol may accelerate the aging of biological components compared to the inert parts of conventional analytical instruments. Enzymatic sensors often exhibit reduced operational stability, requiring recalibration or replacement. In contrast, systems based on molecularly imprinted polymers (MIPs) or aptamers demonstrate increased robustness and satisfactory reproducibility, with coefficients of variation typically remaining within single-digit percentages for field applications [163,166,167,181]. Overall, the evaluation of critical performance characteristics indicates that biosensors are not intended to fully replace reference methods but rather to complement them through in situ, rapid, and cost-effective analysis, thereby enhancing dynamic quality management in the fermentation industry. Table 7 summarizes this comparison by contrasting the main analytical performance characteristics of biosensors with those of established reference methods for ion analysis.

### 4.3. Biorecognition Platforms for Plasticizers and Diphenols

The presence of organic contaminants, such as plasticizers (phthalate esters, PAEs) and bisphenol A (BPA), is an important safety parameter in the fermentation industry [188,189]. These compounds may be intensified by the acidic and ethanolic environment of wine. Due to their documented endocrine-disrupting activity, compliance with established specific migration limits (SMLs) is both a regulatory and quality requirement [3,189]. Analytical detection of these compounds is particularly challenging, as their phenolic structure is similar to natural constituents of must and wine, such as anthocyanins, catechins, and resveratrol [190,191]. This similarity can cause spectroscopic or electrochemical interference in conventional analytical techniques. In this context, the development of selective biosensors offers an alternative approach for rapid and in situ detection, aiming to reduce the need for extensive laboratory procedures.

#### 4.3.1. Biorecognition Elements

The development of biosensors for detecting plasticizers (PAEs) and bisphenols (BPA) in wine faces significant challenges due to the structural similarity of these contaminants to naturally occurring phenolic compounds in the matrix [191]. The phenolic rings of PAEs and BPA resemble those of anthocyanins, resveratrol, and catechins, increasing the risk of non-specific binding or cross-reactive responses [192,193]. Therefore, the success of a biosensor depends primarily on the selectivity of the biorecognition element and its ability to discriminate the target in a chemically complex environment.

Early approaches used oxidative enzymes such as tyrosinase and laccase [194,195]. Although these enzymes have high catalytic activity and relatively low integration cost, their application in wine matrices is limited. Their broad substrate specificity can result in parallel oxidation of natural polyphenols and BPA, increasing the likelihood of false-positive responses. In addition, their proteinaceous nature makes them susceptible to denaturation in the presence of ethanol (>10% *v*/*v*) and under acidic conditions, limiting their operational lifetime. To address these limitations, research has increasingly focused on synthetic and biomimetic receptors [196]. Aptamers, DNA or RNA oligonucleotides selected through the SELEX process, represent a promising approach [197,198]. Through three-dimensional folding, they form specific binding sites for molecules such as BPA or certain phthalate esters, exhibiting high selectivity. Compared to protein-based receptors, aptamers offer greater chemical stability and the ability to refold after exposure to ethanolic environments, although their performance still depends on operational conditions [199].

Molecularly imprinted polymers (MIPs) are of particular interest for industrial monitoring applications [200]. MIPs are synthesized by polymerizing functional monomers in the presence of the target molecule, generating cavities complementary in shape and chemical interaction [201]. Unlike biological systems, they exhibit high chemical and mechanical resistance under acidic pH and in the presence of ethanol. This stability, combined with their potential for low-cost production, makes them especially suitable for developing robust or disposable sensors for monitoring the migration of organic contaminants [202]. Table 8 provides context for this advantage by comparing MIPs with other biorecognition strategies for detecting BPA and phthalates.

#### 4.3.2. Signal Transduction Mechanisms

The binding of the plasticizer or bisphenol to the biorecognition element (MIP or aptamer) constitutes the first stage of the analytical process [199,203]. The next critical challenge is converting this molecular event into a measurable and quantifiable signal, as target concentrations often fall within the ng/L range, corresponding to trace-level detection. Achieving an adequate signal-to-noise ratio under these conditions requires amplification strategies, in which the integration of nanomaterials plays a decisive role [195,204].

Electrochemical transduction is among the most widely applied approaches for developing portable analytical devices, exploiting the ability of phenolic compounds, such as BPA, to undergo electrooxidation at characteristic potentials [205,206]. However, the use of conventional electrodes (e.g., glassy carbon) may be limited by relatively low effective surface area and restricted electron transfer kinetics. To enhance analytical response, electrode surfaces are systematically modified with nanomaterials exhibiting high electrocatalytic activity. Carbon nanotubes (CNTs) and graphene oxide (GO), due to their high surface-to-volume ratio and improved conductivity, increase the electroactive surface and facilitate electron transfer, resulting in enhanced peak currents and improved signal-to-noise ratios [207,208,209]. Additionally, composite materials such as Sonogel-Carbon electrodes have been investigated for increased mechanical stability and reproducibility, features particularly relevant for field applications [210].

Optical transducers offer alternative solutions, particularly for phthalate esters that exhibit limited or less favorable electrochemical activity under mild conditions [211]. Surface plasmon resonance (SPR) technology, combined with MIPs or aptamers, enables real-time monitoring of binding events through changes in refractive index at the sensor surface, without the use of labels (label-free detection). Fluorescence-based systems relying on energy transfer mechanisms (FRET) or quantum dots have also achieved low detection limits [212,213,214]. Nevertheless, their application in red wines requires careful consideration due to the inner filter effect, in which absorption or scattering of radiation by the colored matrix may influence measurement accuracy [191].

Overall, optical methods provide high specificity and sensitivity at low concentrations, whereas electrochemical transduction enhanced with nanostructured carbon materials and screen-printed electrode (SPE) technology offers significant advantages in terms of cost, portability, and reduced susceptibility to optical matrix interference [205]. The selection of an appropriate transduction strategy ultimately depends on the target analyte and the operational requirements of the winemaking process. Table 9 further highlights these trade-offs by comparing the main transduction strategies for BPA and phthalate sensing under fermentation-relevant conditions.

#### 4.3.3. Analytical Validation and Critical Performance Characteristics

The adoption of biosensors as industrial control tools for detecting plasticizers and bisphenols requires comparative evaluation of their performance against established reference methods, such as gas chromatography–mass spectrometry (GC-MS) and liquid chromatography–mass spectrometry (HPLC-MS) [216,217]. Validation procedures primarily focus on sensitivity relative to regulatory limits, selectivity within the complex wine matrix, and operational stability of the sensing platforms.

Regarding sensitivity, modern nanostructured platforms have achieved significant improvements in analytical performance. Incorporating carbon-based nanomaterials or gold nanoparticles has lowered the limits of detection (LOD) of electrochemical and optical biosensors to the nanomolar (nM) range [208,212]. Although GC-MS and HPLC-MS can achieve lower absolute detection limits, the sensitivity achieved by biosensors is generally sufficient for assessing compliance with the Specific Migration Limits (SMLs) defined by European legislation [188]. In this context, biosensors may be considered fit-for-purpose tools for routine monitoring applications, requiring less complex instrumentation and reduced reliance on highly specialized personnel compared to spectrometric techniques.

Managing matrix effects is a central analytical challenge. In red wine, enzyme-based sensors relying on tyrosinase may exhibit limited selectivity due to the oxidation of naturally occurring phenolic compounds structurally similar to bisphenol A (BPA), which can lead to deviations or overestimation of analyte concentrations [205]. In contrast, systems based on molecularly imprinted polymers (MIPs) or aptamers demonstrate increased selectivity toward the target compound [200]. Recovery studies performed in authentic wine samples have reported values between 90% and 110%, indicating satisfactory analytical reliability under acidic and ethanolic conditions [191]. In some cases, this level of selectivity allows for simplified sample preparation protocols compared to chromatographic methods, which typically require extraction and cleanup steps such as solid-phase extraction [217,218].

Operational stability further differentiates available technologies. Enzyme-based electrodes often have limited lifetimes and require controlled storage conditions, whereas MIP- or aptamer-based sensors have been reported to maintain acceptable analytical performance over extended periods under ambient conditions [200]. The enhanced chemical resistance of synthetic recognition elements makes them suitable for on-site monitoring applications. Overall, biosensors are not intended to replace chromatographic reference methods but may serve as complementary rapid screening tools, enabling early identification of potential non-compliant batches before confirmatory laboratory analysis [216]. This complementary role is further supported by the comparison in Table 10, which outlines the main analytical and operational differences between biosensors and chromatographic reference methods for monitoring BPA and phthalates.

### 4.4. Biorecognition Platforms for Key Fermentation Metabolites and Phenolic Constituents

Reliable analytical detection of major fermentation metabolites, such as alcohols, aldehydes, and organic acids, as well as phenolic compounds, within the complex matrix of fermenting must and wine, is essential because these components are primary indicators of fermentation progress and the microbiological stability of the product. Their concentrations also directly affect the organoleptic properties and the management of the winemaking process.

#### 4.4.1. Biorecognition Elements

Most biosensors proposed for detecting key wine fermentation metabolites are based on enzymatic systems, although some approaches using whole cells or synthetic recognition materials have also been investigated [5,12,134].

Ethanol is the main end product of sugar metabolism by yeasts. Consequently, its detection has been the primary target of most biosensors developed for winemaking applications. Most sensors use enzymes such as alcohol dehydrogenase and alcohol oxidase [224,228,229], which catalyze the oxidation of ethanol to acetaldehyde and enable conversion of the biochemical reaction into a measurable electrochemical or optical signal. The widespread use of these enzymes is due to their high substrate specificity and compatibility with various immobilization techniques on electrode surfaces [230,231,232]. Despite their effectiveness, several studies have shown that the stability of enzymatic sensors may be significantly affected by fermentation conditions, such as high ethanol concentrations and pH variations [5,229]. Therefore, various strategies have been developed to improve stability, including enzyme immobilization on nanostructured materials, the use of conductive polymers, or incorporation into hybrid nanostructures that facilitate electron transfer [228,233,234]. These approaches have produced sensors with improved sensitivity and a wider linear measurement range [233,235].

In addition to ethanol, methanol is also of particular interest, as it may form during fermentation mainly through the enzymatic degradation of grape pectins. Although its concentrations in wine are usually low, its presence has significant toxicological implications, making reliable detection important for food safety. Biosensors for methanol are typically also based on enzymes such as alcohol oxidase, which can oxidize small alcohols [236,237]. However, selective discrimination between methanol and ethanol is a major analytical challenge, since these enzymes often show similar activity toward different primary alcohols. Therefore, various strategies have been proposed to improve selectivity, such as using selective membranes or combined enzymatic systems [237]. During fermentation, a series of higher alcohols, such as propanol, isobutanol, and isoamyl alcohol, are also produced. These compounds are mainly formed through the metabolism of amino acids by yeasts and contribute significantly to the aromatic profile of wine. Although their concentration is relatively low compared with ethanol, excessive levels may negatively affect product quality. The development of biosensors for higher alcohols remains limited, mainly due to the lower specificity of available enzymes and the need to discriminate between multiple structurally similar compounds. As a result, analysis of these compounds still relies mainly on chromatographic techniques, while biosensors remain at a relatively early stage of development [5,238].

Acetaldehyde is of particular interest because it is a key intermediate in alcoholic fermentation and affects both the chemical stability and aromatic characteristics of wine. Its detection is more challenging than that of ethanol because it can participate in multiple reactions within the wine matrix. Most biosensors developed for its determination are based on enzymes such as aldehyde dehydrogenase, which enable the selective oxidation of acetaldehyde and convert the reaction into an electrochemical or optical signal [239,240,241]. Despite the satisfactory selectivity offered by this enzyme, several studies indicate that the presence of other carbonyl compounds may affect sensor response, while validation and reporting consistency remain important challenges for broader industrial implementation [5,242].

The detection of organic acids has been the subject of extensive research, as these molecules are directly associated with acidity regulation, microbiological stability, and the progression of malolactic fermentation [243,244]. The main acids targeted by biosensors are malic, lactic, acetic, and tartaric acids. Malic and lactic acids have been studied more extensively because of their role in malolactic fermentation, during which lactic acid bacteria convert malic acid into lactic acid. This process reduces acidity and significantly affects the sensory profile of wine [6,245]. For this reason, several biosensors have been developed based on enzymes such as malate dehydrogenase and lactate dehydrogenase, which enable the selective detection of the corresponding metabolites [246,247,248]. In some cases, bienzymatic systems have also been proposed, allowing simultaneous monitoring of changes in both acids during fermentation [9,249,250].

Acetic acid is an important indicator of microbial spoilage and is associated with the development of volatile acidity in wine. Despite its importance, the development of selective biosensors for acetic acid remains limited. This is partly because acetic acid can arise from multiple metabolic pathways and often coexists with other volatile compounds that can affect sensor response. As a result, several approaches rely on microbial biosensors that exploit the metabolic response of cells as an indicator of the presence of acetic acid [251]. Tartaric acid, although the main organic acid in wine, has been less extensively studied in the context of biosensors. Its relatively stable concentration during fermentation and adequate determination by conventional analytical techniques have limited interest in the development of dedicated biosensors. Nevertheless, some electrochemical approaches based on modified electrodes or conductive polymers have been proposed, although their application in real wine samples still requires further evaluation [243,244,252].

For wine phenolic compounds, the main biorecognition elements are phenol oxidases, especially laccase and tyrosinase, as these enzymes catalyze the oxidation of a wide range of phenolic substrates and enable the development of sensors for estimating either specific phenolic classes or the overall phenolic index [253,254,255]. They offer operational simplicity and good compatibility with electrochemical and optical platforms, but their selectivity is often functional rather than strictly molecular. In complex matrices such as wine, the response often reflects the overall oxidizable phenolic load rather than a single compound [254,256,257]. Nevertheless, enzyme inhibition, activity changes under acidic and ethanol-rich conditions, and limited molecular specificity remain significant analytical constraints for application in real winemaking conditions [5,257,258].

Overall, available data indicate that enzymatic platforms remain the main strategy for detecting both fermentation metabolites and phenolic compounds, mainly because of the high functional selectivity they provide [5,253]. However, issues such as enzyme stability, interference from the complex wine matrix, and the need for long-term sensor operation remain major challenges for broader implementation in real winemaking conditions. Table 11 demonstrates this pattern by comparing the main biorecognition strategies used to monitor major fermentation metabolites and phenolic compounds.

#### 4.4.2. Signal Transduction Mechanisms

For compounds associated with wine fermentation, including key fermentation metabolites and phenolic constituents, selecting an appropriate signal transduction technology is especially important. Biosensors used in oenological systems must operate within a highly complex chemical environment characterized by low pH, increasing ethanol concentration, and the presence of organic acids, polyphenols, pigments, and other electroactive or optically active species that can significantly influence analytical response. Therefore, the development of biosensors for oenological applications relies mainly on electrochemical and optical signal transduction techniques, while conductometric and piezoelectric approaches have been explored to a lesser extent [5].

Among available configurations, electrochemical biosensors are most widely used for detecting fermentation metabolites such as ethanol, acetaldehyde, and organic acids, as well as for monitoring phenolic compounds. Their popularity is primarily due to their high analytical sensitivity, potential for device miniaturization, and relatively straightforward integration into portable or on-site analytical platforms [5,228,229]. In these systems, the enzymatic reaction—or, in some cases, the direct electrooxidation of the analyte—generates or consumes electroactive species that can be detected using amperometric, potentiometric, or voltammetric techniques.

For ethanol detection, the catalytic action of alcohol oxidase produces hydrogen peroxide, which is then oxidized electrochemically at the electrode surface, generating a signal proportional to substrate concentration [224,261]. Similarly, for phenolic systems, enzymatic oxidation catalyzed by tyrosinase or laccase, as well as the direct electrooxidation of certain phenolic compounds, can be converted into amperometric or voltammetric responses [224,262]. In these platforms, carbon electrodes, carbon nanotubes, and graphene-based materials are widely used due to their high conductivity, large active surface area, and the ability to tailor their surface chemistry to improve electron-transfer efficiency and analytical performance [134,263].

Despite these advantages, applying electrochemical sensors to real wine samples presents several analytical challenges. The presence of polyphenols, pigments, and other electroactive compounds can cause undesirable reactions at the electrode surface, leading to electrode fouling, progressive sensitivity loss, and calibration drift. This issue is particularly significant in phenolic sensors, as the oxidation products of phenolic compounds can form passivating layers on the electrode surface, limiting long-term signal stability and repeatability [258,264,265]. To address these effects, many studies have proposed using modified electrodes, such as carbon electrodes functionalized with metal nanoparticles, conductive polymers, or hybrid nanomaterials. These modifications aim to enhance electron-transfer kinetics, increase the effective electroactive surface area, and reduce matrix-related interference effects [137,232]. Optical biosensors provide an alternative method for detecting fermentation metabolites and phenolic compounds. These systems rely on changes in fluorescence, absorbance, or other spectroscopic properties resulting from enzymatic or chemical interactions with the target analyte. A common example is monitoring the conversion of NAD^+^ to NADH in reactions catalyzed by dehydrogenases, as NADH displays distinctive absorbance in the ultraviolet region and serves as an indirect spectroscopic indicator of analyte concentration [266].

Optical platforms have also been developed for phenolic compounds, utilizing changes in absorbance or fluorescence signals. These methods offer high analytical sensitivity and eliminate the need for direct electrochemical contact with the sample. However, their practical use in wine is limited because the wine matrix—especially in red wines—exhibits strong light absorption and scattering due to pigments and polyphenolic compounds [267,268]. These matrix effects can significantly lower the signal-to-noise ratio and complicate quantitative analysis. Additionally, the higher instrumental cost and methodological complexity of optical systems often make electrochemical or hybrid sensing platforms more appealing for practical monitoring during fermentation [269,270].

Other signal transduction mechanisms have also been investigated, including conductometric and piezoelectric sensors, though their use in oenological systems remains limited [5]. Conductometric sensors detect changes in the electrical conductivity of the medium caused by the analytical reaction, while piezoelectric sensors—such as quartz crystal microbalance devices—measure variations in mass at the sensor surface upon analyte binding. Although these methods have shown promising results under controlled laboratory conditions, their application in real fermentation environments is constrained by limitations in sensitivity, selectivity, and operational stability within the chemically complex wine matrix [271,272]. The selection of an appropriate signal transduction mechanism for detecting fermentation metabolites and phenolic compounds depends on the target analyte, required analytical sensitivity, and the physicochemical constraints of the wine matrix. Currently, electrochemical transduction is the predominant approach, primarily due to its high sensitivity, relatively low cost, and compatibility with portable analytical systems. However, its reliability in real wine samples still depends on the choice of electrode materials, effective antifouling strategies, and robust sensor design—factors that ultimately determine the feasibility of broader biosensor implementation in the wine industry. Table 12 further illustrates these considerations by comparing the main signal transduction strategies used to detect fermentation metabolites and phenolic compounds.

#### 4.4.3. Analytical Validation and Critical Performance Characteristics

The transition of biosensors from laboratory development to applications for monitoring the winemaking process requires rigorous evaluation of their analytical reliability. This evaluation is typically conducted by comparing biosensor results with established reference analytical techniques, such as GC and HPLC, which are the principal methods for determining alcohols, carbonyl compounds, and organic acids in wine. For phenolic compounds, additional reference methods include HPLC-UV, HPLC-DAD, and spectroscopic techniques such as UV-Vis, FT-MIR, and FT-NIR coupled with chemometric processing, particularly when the goal is rapid estimation of total phenolic load, anthocyanins, or the evolution of phenolic composition during fermentation [275,276,277,278,279].

Although these methods provide high accuracy and repeatability, their real-time implementation during fermentation is limited by the need for extensive sample preparation and specialized laboratory instrumentation. In this context, biosensors are primarily evaluated as tools for rapid process monitoring, providing complementary information to conventional analytical techniques. For phenolic compounds, the literature is already relatively mature, as electrochemical, optical, and multisensor platforms have been developed for on-site, at-line, and, in some cases, in-line winery applications, aiming to monitor color, total polyphenols, and phenolic evolution during vinification [7,280,281,282].

One of the most important performance characteristics of biosensors is the limit of detection (LOD) and the associated linear response range. For metabolites such as ethanol, sensitivity requirements are less stringent, as its concentration in wine is typically 10–15% *v*/*v*. In contrast, compounds such as methanol, acetaldehyde, and certain higher alcohols are present at much lower concentrations, requiring sensors with higher sensitivity and lower detection limits. Most contemporary enzymatic platforms report LODs in the μM to low mM range for organic metabolites, which are generally sufficient for fermentation monitoring applications [5]. For phenolic compounds, however, evaluation is more complex, since many sensors do not target a single molecule but rather an overall or semi-selective phenolic signal. Accordingly, their performance is judged not only by a low LOD, but also by how reliably the response reflects indices such as total phenolic content, antioxidant capacity, or changes in specific phenolic groups during vinification [253,254,260,283,284].

A second critical criterion is selectivity, the ability of the sensor to distinguish the target analyte from other chemically related compounds. This requirement is especially important for alcohols, as enzymatic sensors based on alcohol oxidases or dehydrogenases may also show activity toward other primary alcohols, such as methanol or certain higher alcohols [238,262,272]. Similarly, for organic acids, the presence of structurally related carboxylic acids may affect sensor response. For phenolics, selectivity is even more challenging because wine contains a highly complex mixture of anthocyanins, flavonoids, phenolic acids, and tannins, each with distinct electrochemical and enzymatic behavior [281,284]. As a result, many phenolic sensors serve more as tools for estimating the overall phenolic index or total antioxidant activity than as strictly molecularly selective systems. For this reason, selectivity is usually evaluated through interference studies and comparison with chromatographic or spectroscopic reference methods [253,256].

Several studies have reported recovery values between 85% and 110%, which are generally considered acceptable for process-monitoring applications [230,231]. For phenolic compounds, matrix effects are often even more pronounced, as the strong absorbance of the wine matrix in the ultraviolet and visible regions, the overlap of spectral signals, and the tendency of oxidized phenolics to adsorb onto electrode surfaces may affect both optical and electrochemical measurements. Therefore, the use of chemometric tools, calibration in real matrices, and the application of antifouling surface strategies are critical components of analytical validation [264,265,277,278,285,286,287].

In addition to analytical accuracy, operational stability is also a major criterion. Enzymatic biosensors may gradually deactivate due to pH fluctuations, the presence of organic solvents, or thermal degradation of proteins [287]. In fermentation applications, where ethanol concentration progressively increases, enzyme stability becomes a critical factor for maintaining measurement reliability. Consequently, many recent studies have focused on immobilization strategies that enable the reuse of sensors over many measurement cycles [263,288]. Similarly, in phenolic sensors, stability depends not only on the resilience of the recognition biomolecule but also on the platform’s ability to limit surface passivation caused by oxidation products of phenolic compounds. In this context, nanomaterials, modified carbon electrodes, and stable enzyme-immobilization platforms have substantially improved repeatability and operational stability, enhancing the usefulness of such sensors for at-line or on-site winery applications [257,289].

Overall, available data indicate that biosensors can provide reliable information for monitoring key fermentation metabolites, particularly when used as tools for rapid assessment of process progression. For phenolic compounds, the outlook is even more promising for practical implementation, as the literature documents several optical, electrochemical, and multisensor platforms designed for real winery applications, especially for monitoring color, total polyphenols, antioxidant status, and phenolic evolution during fermentation [280,281,289]. Nevertheless, the complete replacement of established chromatographic techniques remains limited, mainly due to issues related to selectivity, stability, and matrix effects. Biosensors should therefore currently be regarded more as complementary tools for process monitoring than as full alternatives to reference analytical methods. This perspective is summarized in Table 13, which presents the analytical performance of biosensors compared to established methods for monitoring key fermentation metabolites and phenolic compounds.

### 4.5. Integration of Biosensors into HACCP Planning

Recognizing vessel–product interactions as CCPs, in accordance with HACCP principles and as discussed in Section 3.3, is essential regardless of vessel construction material, as these systems remain in prolonged and direct contact with the fermenting matrix under acidic and ethanolic conditions. The objective at this stage is not to reiterate the associated hazards, but to define how the previously discussed biosensors can be operationally integrated into a structured HACCP framework [290].

For stainless steel tanks, where the CCP involves potential corrosion and release of metallic ions, metal-specific biosensors (MIP- or aptamer-based platforms) can be incorporated into the CCP monitoring phase during active fermentation [291]. Periodic or continuous measurements allow comparison with predefined critical limits or internal alert thresholds and enable timely corrective actions in case of deviation [292]. In ceramic fermentation vessels, where the primary concern is possible heavy metal migration from glazed surfaces, DNAzyme- and aptamer-based sensors can function as in-process monitoring tools [108,110]. Their role is not to replace initial material certification but to provide ongoing surveillance during use, particularly in artisanal or non-standardized constructions where glaze stability may vary.

Concrete and cementitious tanks introduce CCPs related to alterations in ionic composition and potential leaching of inorganic constituents. In this case, ion-selective biosensors may serve both safety and process monitoring functions, as changes in ionic balance can affect fermentation kinetics and regulatory compliance [293]. Plastic fermentation vessels present CCPs associated with possible migration of organic additives or residual monomers under ethanolic conditions [188]. Biosensors targeting organic contaminants, particularly MIP-based or nano-enhanced electrochemical platforms, can be applied as in-process monitoring tools to assess material stability throughout fermentation [200,205].

In addition to direct hazard monitoring, biosensors targeting key fermentation metabolites and phenolic indicators provide complementary process-level information within the HACCP framework. Variations in ethanol, organic acids, acetaldehyde, and selected phenolic parameters do not necessarily define vessel-related CCPs on their own, but they can serve as early indicators of process disturbances, altered fermentation kinetics, oxidative instability, or matrix changes associated with vessel–product interactions. In this context, these biosensors enhance HACCP monitoring not as primary hazard-specific tools, but as supportive indicators of process deviation and product quality deterioration during fermentation.

Across all vessel types, biosensors are positioned within the monitoring stage of HACCP, while reference analytical methods (ICP-MS, GC-MS, HPLC-MS) retain their role in verification [217,294]. Their combined application shifts vessel-related CCPs from static post-process verification points to actively controlled process parameters during alcoholic fermentation. Consequently, the structured adaptation of biosensors to identified CCPs strengthens the preventive dimension of HACCP, enabling early deviation detection and dynamic risk management throughout fermentation. Table 14 applies this HACCP-oriented approach by mapping biosensor platforms to specific CCP monitoring needs for various fermentation vessel types.

## 5. Applications Under Real Fermentation Conditions

The use of biosensors in real fermentation conditions marks a crucial shift from laboratory validation to practical industrial integration in winemaking processes. Unlike controlled laboratory settings, alcoholic fermentation takes place in a dynamic, chemically complex, and challenging environment, where acidity, rising ethanol levels, polyphenolic content, and ongoing compositional changes directly affect the thermodynamic stability and analytical reliability of sensing platforms. Therefore, evaluating biosensor technologies requires more than assessing their metrological performance; it must also address the strategic implementation method (in situ or at-line), compatibility with HACCP-based control systems, and the development of technological adaptations that ensure robustness, reproducibility, and economic viability. This section synthesizes these aspects, highlighting both the practical potential and the realistic limitations of biosensor use under actual fermentation conditions.

### 5.1. Implementation Strategies and Technological Maturity

The integration of biosensors into the wine production line marks a shift from laboratory documentation and validation to industrial application, aiming to support real-time decisions or decisions at critical control points. In this context, the choice of analytical strategy depends not only on the metrological performance of the device but, more importantly, on the operational value of the information—specifically, when it is needed, how often, and for which decision. Literature and industrial practice identify two main operating models: in situ monitoring, where measurement is conducted directly or via a bypass flow within or next to the tank for dynamic process control, and at-line control, where measurement is performed through rapid, sampling-based checks near the production line, focusing on compliance and safety. The suitability of each model depends on the analyte category (technological ions, transition metals, toxic contaminants), the technological maturity level (TRL) of the corresponding sensors, and the constraints imposed by the wine matrix on operating conditions. In this context, it is important to distinguish between analytes that support dynamic process control, such as alcohols, organic acids, and acetaldehyde, and analytes that indicate changes in other key wine constituents considered critical quality factors, such as selected phenolic indicators and color-related parameters. The value of each measurement depends not only on analytical sensitivity but also on whether the information is needed continuously, periodically, or at specific control points during fermentation [5,7,253,275].

#### 5.1.1. In Situ Applications

The application of in situ biosensors—meaning their direct immersion in, or connection via a bypass loop to, the fermenting must—aligns with the philosophy of Process Analytical Technology (PAT) [216]. The practical viability of this approach depends primarily on the nature of the analyte, its electrochemical behavior, and the physicochemical resilience of the sensor under conditions of low pH, increasing ethanol concentration, and high organic load.

For technologically relevant metal ions (K^+^, Ca^2+^), technological maturity is high, as measurement is based on potentiometry using ion-selective electrodes (ISEs) [293]. These sensors operate according to the Nernst equation, measuring a potential difference related to ion activity without consuming the analyte or requiring preconcentration [296]. At the industrial level, flow setups installed on bypass pipelines enable continuous monitoring of potassium activity, which is directly linked to the saturation temperature (Tsat) of potassium bitartrate [297]. This information can be used for targeted control of cooling during stabilization, reducing energy consumption and avoiding excessive cooling.

Biosensors targeting key fermentation metabolites such as ethanol, malic acid, and lactic acid are well suited for in situ or semi-continuous monitoring using bypass configurations, as these parameters change dynamically and are directly linked to the kinetics of alcoholic and malolactic fermentation [11]. In this context, the operational value of measurement is high, since continuous or semi-continuous monitoring enables early detection of delayed or abnormal fermentation, optimization of acidification or deacidification strategies, and assessment of malolactic conversion progress. Electrochemical bienzymatic biosensors for malic and lactic acid have already demonstrated practical suitability for on-site process monitoring, while ethanol is also a mature analytical target for continuous or near-continuous tracking of fermentation progress [247,250].

For transition metals such as iron, the use of appropriately modified electrodes enables discrimination between Fe^2+^ and Fe^3+^ forms [292]. This distinction is operationally important, as Fe^2+^ actively participates in redox reactions that affect wine stability and its aromatic profile. In situ monitoring of such changes supports the control of micro-oxygenation and the adjustment of oxidative conditions during fermentation [298]. For phenolic compounds, in situ implementation is technically more demanding, mainly because of strong matrix absorbance, turbidity, and the tendency of oxidation products to passivate electrochemical surfaces. Nevertheless, several in-line or bypass optical and electrochemical methods have been developed for monitoring color development, phenolic extraction, and the overall phenolic signal during red winemaking. This shows that this category of analytes can be integrated into semi-continuous monitoring schemes when the goal is to track quality evolution rather than to achieve strict quantitative determination of individual compounds [7,278,281].

In contrast, in situ detection of heavy metals (Pb^2+^, Cd^2+^) presents significant technical limitations. The most sensitive electrochemical technique for trace-level detection, anodic stripping voltammetry (ASV), requires a preconcentration step under negative potential [132]. In the must matrix, the electrode surface is simultaneously exposed to polyphenols, proteins, and colloids, which adsorb rapidly and cause passivation [135]. The resulting organic film increases charge-transfer resistance and reduces the peak current within a few measurement cycles, degrading stability and repeatability. The need for frequent electrochemical regeneration or replacement of electrodes increases complexity and operating cost, making continuous in situ implementation of limited practical value in an industrial environment [299,300].

The category of organic contaminants (bisphenol A and phthalate esters) shows even lower compatibility with in situ monitoring. Sensors in this group rely on affinity mechanisms, using aptamers, antibodies, or molecularly imprinted polymers [199,200]. Target binding is often accompanied by receptor conformational rearrangement, while regeneration requires specialized buffer solutions with controlled pH or ionic strength, which are not compatible with direct use inside the fermentation tank. In addition, the kinetic migration of plasticizers from polymeric materials is described by diffusion coefficients on the order of D ≈ 10^−10^–10^−12^ m^2^/s, implying slow concentration changes over days or weeks [188]. Under these conditions, the requirement for continuous real-time monitoring is not operationally justified.

Overall, the in situ approach is highly suitable for technologically relevant ions and certain transition metals, where measurement can be performed without preconcentration steps and with robust electrochemical mechanisms. By contrast, for trace elements that require preconcentration and for organic contaminants relying on affinity-based sensors, the technical complexity and the kinetics of the phenomenon render in situ application of limited practical value.

#### 5.1.2. At-Line Applications

Unlike the continuous data flow of in situ monitoring, at-line analysis relies on targeted sampling at critical time points in the production process, using portable or benchtop devices placed near the production line [216]. This approach enables the use of highly sensitive nano-biosensors without the limitations caused by prolonged sensor exposure to the fermenting medium. As a result, it provides a practical solution for safety and compliance controls [301,302]. The at-line approach is particularly suitable for monitoring phenolic parameters, as it allows controlled sampling, reduces matrix-related interferences, and enables the use of rapid optical or electrochemical devices near the production line. In practice, this is especially useful for tracking color development, total polyphenols, antioxidant status, and phenolic evolution during fermentation—parameters that do not necessarily require continuous sensor immersion but benefit from frequent monitoring at predefined checkpoints. At the same time, biosensors targeting key fermentation metabolites, such as ethanol, organic acids, and acetaldehyde, can also operate effectively within at-line schemes, particularly when continuous exposure of the sensor to the fermentation matrix is not operationally necessary or may disproportionately affect the stability of the sensing platform [7,11,280,281,282].

For metal ions of technological interest, at-line measurement complements in situ monitoring by providing a mechanism for verification and calibration. The use of portable ion-selective analyzers to determine the activity of Ca^2+^ and Cu^2+^ allows more accurate calculation of treatment additions (e.g., bentonite, stabilizing agents), reducing overdosing that often results from empirical practices. Particularly for copper, at-line measurement before bottling is critical for avoiding haze phenomena (cupric casse), as it allows estimation of the free and reactive forms of the metal, which are not always adequately captured in total concentration measurements [173,292].

The category of heavy metals (Pb^2+^, Cd^2+^) finds the at-line model most suitable for application. The use of disposable screen-printed electrodes (SPEs), modified with bismuth or gold nanoparticles, addresses the problem of surface passivation that limits in situ configurations [107,295,303,304]. The single-use nature limits memory effects (carry-over) and the accumulation of contaminants. In addition, the at-line procedure allows controlled and minimal sample pretreatment, reducing matrix effects and optimizing ASV performance. In this way, these sensors can function as screening tools at raw material reception, enabling immediate exclusion of batches that exceed maximum permissible limits before they enter the production process [132,133,304].

For organic contaminants and plasticizers (bisphenol A, phthalate esters), the at-line approach is essentially the only operationally viable compliance control strategy. Migration of these compounds from contact materials is cumulative and relatively slow; therefore, control focuses on specific points such as before bottling or at the batch release stage [188]. Biomimetic systems based on molecularly imprinted polymers (MIPs) or aptamers have been integrated into portable electrochemical readers or rapid screening devices, providing “in/out-of-limit” results within a short time [199,200,203]. Although standardization for the wine matrix is still evolving, their at-line application significantly reduces cost and time compared with outsourced chromatographic analysis. Overall, the at-line strategy offers advantages in analytical stability, matrix control, and economic viability, particularly for parameters that do not require continuous real-time monitoring [134].

#### 5.1.3. Comparative Evaluation and Selection Criteria

The choice between in situ and at-line strategies is not merely a technical decision but results from integrating sensor technological maturity, the operational value of the information, and the nature of the analytical target. The final selection depends on three interrelated factors: (a) the sensor’s level of technological maturity (TRL), which directly affects reliability and the feasibility of integration into the production line; (b) the requirement for continuous versus periodic information, that is, whether the measurement supports dynamic process control or control at predefined checkpoints; and (c) the tolerance of the wine matrix and the degree of sample pre-treatment needed to ensure stability, repeatability, and an acceptable level of interference [305,306].

In situ monitoring is preferred when the objective is automation and immediate responsiveness to parameters that change dynamically during fermentation and influence process evolution. However, its practical implementation involves higher installation costs and increased maintenance demands due to biofouling, variations in conductivity and viscosity, and, more generally, the continuous exposure of the sensor to a low-pH environment with increasing ethanol content and high organic load. In contrast, the at-line approach relies on sampling but offers significant advantages in analytical stability, lower cost per analysis, and more effective management of interferences through minimal and controlled sample pre-treatment [307,308,309].

Based on these considerations, a more refined selection framework can be established. Parameters that vary rapidly and are directly linked to fermentation kinetics and technological process control—such as ethanol, organic acids, acetaldehyde, and certain technologically relevant ions, including K^+^, Ca^2+^, Fe^2+^/Fe^3+^, and Cu^2+^—are generally better suited to in situ, bypass, or semi-continuous monitoring configurations, where immediate analytical feedback supports timely corrective interventions. In contrast, phenolic indicators, although critically important for wine quality evolution, color development, and oxidative stability, are more commonly incorporated into at-line or hybrid monitoring schemes, where controlled sampling improves analytical robustness and reduces matrix-related interferences. Parameters primarily associated with chemical safety and regulatory compliance, such as Pb^2+^, Cd^2+^, As, bisphenol A, and phthalate esters, are more appropriately addressed through at-line control strategies, as these require higher analytical reliability, minimization of interference effects, and verification at defined control stages, including raw material reception, pre-bottling, and batch release. The combined use of both strategies enables a balance between technological innovation, economic feasibility, and quality assurance, establishing a realistic framework for integrating biosensors into modern systems for monitoring and controlling winemaking processes. This proposed allocation of analytes to various monitoring configurations is summarized in Table 15, which outlines practical deployment strategies and decision-making pathways for biosensor-based monitoring in wine fermentation.

### 5.2. Operational Limitations and the Impact of the Wine Matrix

The transition of biosensors from controlled laboratory conditions to real winemaking environments presents significant technical challenges due to the complexity of the wine matrix. Must is a dynamic, chemically evolving system in which ethanol, organic acids, sugars, inorganic ions, polyphenols, and colloidal macromolecules coexist. During fermentation, the concentrations and interactions of these components change continuously, directly affecting the operational stability of biosensors. The main mechanisms limiting the reliability of real-time measurements can be classified into three categories: (a) biofouling and surface passivation, (b) electrochemical and spectroscopic interferences, and (c) physicochemical incompatibility of biorecognition elements with the hydroalcoholic environment.

#### 5.2.1. Biofouling and Passivation Phenomena

Biofouling is the most significant limiting factor for in situ applications. Introducing an artificial surface into the fermenting medium immediately triggers spontaneous adsorption processes [182,216]. Within seconds of immersion, an initial “conditioning film” forms, consisting of small organic molecules and ions. This is followed by competitive adsorption of macromolecules, mainly proteins, yeast enzymes, and polysaccharides (e.g., pectins, glucans) [180]. These processes are driven by hydrophobic interactions, electrostatic forces, and van der Waals forces. The result is the gradual coverage of the sensor’s active surface, sometimes irreversibly [180,300]. In electrochemical configurations, the adsorbed layer increases the charge transfer resistance (Rct) at the electrode–solution interface and restricts analyte diffusion toward the recognition surface. This leads to decreased response current, increased response time, and progressive signal drift [134].

Alongside physical blocking, the wine matrix also causes chemical passivation of electrodes, mainly due to the high concentration of phenolic compounds [269]. When anodic potential is applied, phenolic compounds such as catechins and caffeic acid are oxidized to reactive phenoxyl radicals. These radicals polymerize on the electrode surface, forming a compact, electrically insulating polymeric film [135,310]. This surface insulation particularly affects amperometric techniques and methods such as anodic stripping voltammetry (ASV) for detecting Pb^2+^ and Cd^2+^ [132]. The insulating layer reduces the peak current and alters the linearity of the calibration curve, making measurements unstable unless systematic cleaning or surface renewal protocols are applied.

Beyond organic deposits, in situ sensors are also subject to inorganic scaling. Must and wine are often supersaturated with respect to potassium bitartrate (KHT). Micro-defects on the surface of ion-selective electrode membranes can serve as sites of heterogeneous nucleation, promoting crystal growth [177,311]. Crystalline deposition alters the local ionic balance at the interface and causes significant potential drift. Removing these deposits requires chemical treatment with acidic or alkaline solutions, a process that interrupts continuous operation and reduces the sensor’s operational lifetime [134,312]. Consequently, biofouling, polyphenolic passivation, and inorganic scaling are critical factors that limit the reliability and long-term stability of in situ systems in fermentation environments.

#### 5.2.2. Impact of the Wine Matrix

The heterogeneous and dynamically changing composition of the wine matrix significantly affects the analytical reliability of biosensors. The so-called “matrix effect” is not limited to a simple increase in noise but also alters the chemical form (speciation) of analytes, their activity, and the kinetics of recognition processes [313,314]. These effects are especially important in electrochemical and affinity-based sensors, where the response depends on the free form of the analyte and the local conditions at the interface [315]. A fundamental limitation in potentiometric and amperometric detection of metal ions such as Cu^2+^ and Fe^3+^ is distinguishing between total concentration and the free ionic form [316]. Must contains high concentrations of organic acids (tartaric, malic), phenolic compounds, and other complexing agents (ligands) [171]. Transition metals form thermodynamically stable complexes with these compounds, greatly reducing the fraction of free ions in solution.

Ion-selective electrodes (ISEs) and many electrochemical techniques respond to the activity of the free ion rather than the total concentration [177]. As a result, a negative bias is often observed compared to total analysis techniques such as ICP-MS [101]. Although this constitutes an analytical discrepancy, from a technological perspective, measurement of the bioavailable form may be more relevant to phenomena such as oxidative reactions, haze formation, or catalytic processes. Therefore, the matrix effect is not only a source of error but also a factor that differentiates the type of information provided by the sensor. Electrochemical detection of trace levels of heavy metals (Pb^2+^, Cd^2+^) in the wine matrix is affected by the strong redox background of the system [135]. Many phenolic constituents, such as catechins and hydroxycinnamic acids, are electroactive and exhibit oxidation potentials that overlap with or are close to those of the target metals [310]. During potential scanning, the faradaic currents associated with oxidation of these organic molecules increase the background and reduce the signal-to-noise ratio (SNR) [186]. Peak overlap hinders accurate quantification, especially in techniques such as anodic stripping voltammetry (ASV) [132].

Simultaneously, adsorption of organic compounds on the electrode surface alters the double-layer capacitance, leading to baseline shift and distortion of the electrochemical response [135]. These changes are not constant during fermentation, as the concentration and structure of phenolics change dynamically. For organic contaminants such as bisphenol A and phthalate esters, sensors rely on affinity mechanisms (aptamers, antibodies, MIPs) [199,200]. The selectivity of these systems depends on spatial and chemical complementarity between target and receptor. The wine matrix contains numerous natural compounds with aromatic rings and phenolic groups, which may exhibit structural similarity to the target analytes [317,318]. Competitive binding of these components to recognition cavities leads to cross-reactivity and possible false-positive results.

In addition, changes in the dielectric constant of the medium due to increasing ethanol and ionic strength affect the affinity constant (Kaff) and the binding and unbinding kinetics (kon/koff) [227,319]. Variation in these parameters can reduce specificity and prolong response time, particularly in systems not designed specifically for a hydroalcoholic environment. Overall, the impact of the wine matrix is not merely a technical nuisance but a fundamental constraint that differentiates the analytical behavior of biosensors under real conditions. Understanding the mechanisms of complexation, electrochemical overlap, and cross-reactivity is necessary for correct interpretation of data and rational design of calibration and validation strategies.

#### 5.2.3. Environmental Incompatibility

Reliable operation of biosensors under real winemaking conditions requires thermodynamic stability of the recognition elements and stable charge and mass transport at the interface [271]. Must and wine are chemically complex hydroalcoholic systems with low pH, varying ionic strength, and increasing ethanol concentration—factors that directly affect the functionality of both biological and synthetic sensors [320].

The acidity of the medium (pH 3.0–3.8) deviates substantially from the physiological operating range of many biological receptors. Increased proton concentration alters the ionic state of amino acid residues in enzymes and antibodies, affecting tertiary structure and the affinity constant (Kaff). Changes in protonation may reduce binding capacity or cause partial unfolding of the protein structure, resulting in loss of sensitivity and repeatability [321]. Similar effects occur in synthetic systems. In Molecularly Imprinted Polymers (MIPs), acidity modifies the ionic state of functional groups involved in binding, affecting adsorption equilibrium [200]. In electrochemical devices, pH variations can shift peak potential and change the shape of voltammetric curves, especially when the reaction is proton-dependent [322]. Ethanol, at concentrations up to 15% *v*/*v*, alters solvent properties such as dielectric constant and water activity. These changes affect electrostatic interactions and biomolecular stability. In biological receptors, ethanol disrupts the hydration shell and can destabilize protein structure, influencing binding kinetics [323]. In polymeric systems, such as PVC membranes of ion-selective electrodes or MIPs for detecting organic contaminants, ethanol can penetrate the polymer network and induce swelling [227,324,325]. Swelling alters the geometry of recognition cavities and diffusion conditions, leading to loss of selectivity, changes in sensitivity, and increased response time. Additionally, changes in the dielectric constant of the medium affect complexation and affinity equilibrium constants, making calibration in a hydroalcoholic environment—rather than in aqueous standards—necessary [227].

Fermentation is a dynamic system with intense carbon dioxide production and changes in medium flow. The release of CO_2_ bubbles creates local disturbances in the diffusion layer at the sensor surface [326]. These fluctuations transiently alter mass transport and cause unstable signals or signal spiking, particularly in amperometric devices. At the same time, low dissolved oxygen during the anaerobic stage of fermentation limits the applicability of detection mechanisms based on O_2_-dependent oxidative reactions [327]. Hydrodynamic instability and viscosity changes affect the stability of mass transport, reducing repeatability in in situ measurements [328]. Overall, the environmental incompatibility of the wine matrix results from a combination of low pH, hydroalcoholic character, and dynamic hydrodynamic behavior. These factors constrain the thermodynamic and kinetic stability of biosensors, making it necessary to adapt the platform to this specific environment or shift certain applications toward at-line strategies [329].

### 5.3. Optimization Strategies and Technological Solutions

Overcoming the physicochemical constraints imposed by the wine matrix typically requires redesigning the sensing architecture rather than incrementally refining conventional configurations. Current technological developments fall into three main categories: (i) reinforcement and protection of the sensing interface, (ii) replacement of vulnerable biological recognition elements with biomimetic systems, and (iii) integration of automated microscale sample conditioning before detection. The underlying approach is sequential: interface protection, enhancement of platform robustness, and control of the local sensing environment [290,330].

Reduced sensitivity and slow electron-transfer kinetics under acidic conditions are commonly addressed with high-activity nanostructured interfaces [210,331]. In oenological applications, another priority is replacing hazardous materials (e.g., mercury) with environmentally compatible alternatives. For trace Pb and Cd determination at low pH, bismuth-film electrodes are widely used because they form intermetallic deposits with target analytes, enabling peak discrimination while minimizing interference from hydrogen evolution currents that intensify in acidic must [332]. Further integration of carbon nanotubes or graphene increases the electroactive surface area and improves charge-transfer efficiency, allowing detection at potentials where polyphenol-related interference is reduced [133,333]. Matrix-induced passivation by organic acids and polyphenols is often mitigated using selective membranes, with Nafion as a representative example [133,334]. Its sulfonated functionality provides electrostatic exclusion of anionic species (e.g., tartrate, malate, ascorbate) while permitting cation transport to the electrode surface. To reduce adsorption of proteins and polysaccharides, hydrophilic coatings such as polyethylene glycol layers or hydrogel films are used to form hydrated barriers that limit nonspecific deposition [335]. This approach prioritizes fouling prevention through interface engineering rather than continuous electrode cleaning.

Low pH and increasing ethanol concentrations can compromise the thermodynamic stability of enzymes and antibodies. As a result, the transition to synthetic and biomimetic recognition elements is increasingly treated as a design requirement rather than an optional alternative [336]. Molecularly imprinted polymers (MIPs) function as “synthetic antibody” analogs by incorporating rigid binding cavities complementary in geometry and functional group distribution to the target analyte. Due to high crosslink density, MIPs retain structural integrity at pH values below 3 and under elevated ethanol content where protein receptors may denature [164,166,200]. Recognition relies primarily on physicochemical complementarity rather than tertiary protein structure, supporting their use for plasticizers and small organic contaminants without reliance on complex buffering conditions [337]. Aptamers provide an additional advantage through reversible folding behavior. Unlike proteins, nucleic acid receptors can undergo reversible conformational transitions and recover binding functionality under restored conditions, which is beneficial under fluctuating pH and temperature [112,338]. Chemical functionalization with electroactive labels enables direct signal transduction without enzymatic amplification steps, supporting the development of reusable sensors with extended operational lifetimes compared to conventional immunosensors [339].

Direct immersion of sensors into fermenting must imposes inherent constraints associated with matrix variability. Microfluidic platforms address this by controlling the local sensing environment through precise handling of small volumes and programmable adjustment of pH, ionic strength, and dilution before measurement, without disturbing the main fermentation vessel [340]. This provides a practical way to mitigate pH incompatibility and matrix-driven variability at the sensing interface. In contrast to static systems, microfluidic architectures can incorporate automated washing and recalibration cycles, reducing baseline drift and enabling multi-day or multi-week operation with minimal manual intervention. Integration with wireless data transmission further supports real-time monitoring and linkage to process control systems, positioning the biosensor not only as an analytical device but also as a component of quality and risk management within fermentation operations [341].

## 6. Regulatory Framework and Food-Contact Safety in Fermentation Systems Integrating Biosensors

Ensuring wine quality and safety depends not only on fermentation biochemistry but also on the chemical behavior of materials that come into contact with must or wine. Fermentation vessels (stainless steel, ceramics, polymers, concrete, or linings) and any immersed or adjacent monitoring device (such as in situ probes, sensing heads, membranes, adhesives, or coatings) are considered Food Contact Materials (FCMs) and must comply with strict safety requirements. The core principle is that materials must not transfer constituents into the product at levels that endanger human health or cause unacceptable changes in composition or organoleptic properties—an especially demanding requirement under low pH and increasing ethanol content. In practice, this requires documented chemical inertness, controlled migration behavior, and traceability, with added complexity when “active/intelligent” elements such as biosensors are introduced.

### 6.1. Fermentation Vessel Materials: Harmonization, Limits, and Regulatory Gaps

Within the European Union, the general legal basis is Regulation (EC) No 1935/2004 [3], which sets overarching safety principles and establishes traceability obligations, including the requirement for a Declaration of Compliance where applicable. However, implementation is not uniform across material classes. Plastics and polymeric materials are covered by a harmonized framework (Regulation (EU) No 10/2011 [44]) that includes positive lists of authorized substances and defined limits for specific and overall migration (SML/OML), providing a clear compliance pathway. In contrast, stainless steel and concrete do not have an EU-wide specific measure of comparable detail; compliance is often supported through national provisions, technical recommendations, and the principle of mutual recognition, resulting in regulatory asymmetry across Member States. For ceramics, the EU framework has historically focused on Pb/Cd migration, while broader concerns (such as other trace elements, glaze or coating performance, and sealing systems) are commonly addressed through national practices and product-specific conformity testing.

Internationally, approaches diverge further. In the United States, the [342,343,344] framework evaluates food-contact materials and substances through exposure-based safety demonstration; in China, GB standards often rely on stricter positive lists and mandatory testing [345]. The International Organisation of Vine and Wine (OIV), while not legally binding, remains influential as a technical reference through international codes and recommended practices, particularly regarding “inert” materials and oenological operations [346,347,348]. A key practical implication for fermentation is that compliance depends not only on the material category but also on realistic conditions of use (pH, ethanol, temperature, contact time); therefore, testing strategies must reflect hydroalcoholic exposure rather than idealized aqueous conditions.

### 6.2. Biosensors in Fermentation: From “Instrument” to FCM and the Nanomaterial Issue

Integrating biosensors into fermentation tanks shifts regulatory focus from the passive inertness of the vessel to the functional safety of a continuously immersed device. An in situ sensor is not merely a measurement tool; it is a food-contact assembly comprising substrate materials, electrodes, membranes, coatings, encapsulants, adhesives, and protective housings [3,349,350]. Therefore, it must be assessed from an FCM perspective, where the requirement is not only the absence of harmful chemical release but also the functional containment of active components (such as metals, catalysts, additives, and nanostructures) under mechanical and chemical stress (CO_2_ evolution, agitation, temperature fluctuations, biofouling).

Nanomaterials present a specific regulatory bottleneck. In EU practice, nanoforms are not automatically considered equivalent to their bulk counterparts, even when the bulk substance is known or authorized in food-contact contexts [3,351]. This creates additional risk assessment requirements, particularly when nanostructures are accessible at the interface or when abrasion, delamination, or degradation could lead to their release into the matrix. For fermentation-compatible implementation, this drives a strong “regulatory-to-design” approach: preference for closed or encapsulated architectures (such as protective barriers with controlled analyte permeability) and, when continuous immersion cannot be robustly justified, a shift toward at-line formats where contact time and exposure can be more defensibly controlled and documented.

### 6.3. Regulatory Asymmetry and the Need for Fermentation-Realistic Test Conditions

The central challenge is not a lack of regulation, but the mismatch between rapid technology evolution and standardized testing protocols tailored to fermentation conditions. Two points are critical for winemaking systems:

First, regulatory fragmentation for certain structural materials (notably metals/alloys and concrete/linings) leads to different compliance expectations across jurisdictions, increasing the documentation burden for suppliers and wineries, especially when equipment and sensing devices are intended for international markets.

Second, conventional migration tests are often performed under static conditions, whereas alcoholic fermentation is inherently dynamic: CO_2_ production, changes in viscosity and conductivity, low pH, high organic load, mechanical stress, and surface biofouling. For in situ sensors, this means that beyond baseline migration data, use-condition validation becomes essential: stress testing for coating integrity and delamination, abrasion and fatigue evaluation, and verification that the device does not introduce physical hazards (such as detachment of micro-components). In practice, integration into food safety management systems (HACCP or ISO 22000) must explicitly address these risks alongside chemical migration [352].

Including regulatory analysis in a review of biosensing strategies across wine fermentation vessels is scientifically justified when approached from a “regulatory-to-design” perspective. Vessels and biosensors should be considered a single food-contact system, with requirements for chemical inertness, functional containment, and fermentation-realistic testing under hydroalcoholic and dynamic conditions. In this context, regulation is not an appendix to the technology discussion; it becomes a selection criterion that directly informs platform architecture and deployment strategy (in situ versus at-line) for safe, reliable, and industrially credible fermentation monitoring.

## 7. Conclusions

This review shows that alcoholic fermentation is a dynamic, chemically evolving system in which the interaction of tank material, physicochemical changes (pH, rising ethanol, temperature fluctuations, phenolic and colloidal fractions, CO_2_), and microbial activity collectively shape safety and quality parameters. Available data indicate that no fermentation vessel material—stainless steel, ceramics, cementitious or concrete materials, polymers, or coatings—is fully inert under real winemaking conditions, as mechanisms of inorganic ion or metal release and organic compound migration are observed, with intensity depending on the chemical aggressiveness of the hydroalcoholic matrix and variable process conditions. Therefore, targeted monitoring of technologically critical ions and transition metals, toxic trace elements, and organic migrants or contaminants is an essential component of modern risk management in fermentation, with emphasis on measurable reliability within the actual matrix.

Biosensors represent an important technological direction for moving from intermittent laboratory analyses to operational monitoring with high temporal resolution. However, the choice of strategy depends on the analyte category, required operational information, and technological maturity. In this respect, it is also necessary to distinguish between analytes primarily associated with safety and compliance, analytes directly linked to fermentation kinetics, and analytes that reflect the evolution of product quality. In situ applications align with the philosophy of Process Analytical Technology and are more suitable for process-control parameters that change dynamically and can be measured without pre-concentration. In contrast, for toxic heavy metals that require pre-concentration and for organic pollutants or plasticizers that rely on affinity mechanisms and have slow migration kinetics, continuous in situ monitoring has limited functional value and greater technical complexity. In this context, the at-line model is operationally dominant for compliance and safety control at critical points, offering greater analytical stability and more effective interference management.

At the same time, biosensors should not be viewed solely as tools for monitoring hazardous migrants or contaminants. Biosensors targeting key fermentation metabolites—such as ethanol, organic acids, and acetaldehyde—provide information directly related to fermentation kinetics, malolactic progression, and early detection of process disturbances. Their value therefore extends beyond analytical determination to operational decision support, particularly where rapid or semi-continuous measurements can reveal delayed, deviating, or otherwise suboptimal fermentation behavior before these issues become apparent through conventional laboratory testing. In addition, biosensors addressing phenolic indicators are increasingly relevant for quality-oriented monitoring, as they can provide timely information on color evolution, total phenolic changes, antioxidant status, and extraction- or oxidation-related transformations during fermentation.

Performance limitations under real conditions are mainly due to fouling and biofouling, interferences from polyphenols and proteins, pH and ethanol variability, CO_2_-driven hydrodynamic instability, and calibration drift, all of which affect long-term functional stability and the feasibility of reliable integration. Thus, moving from prototypes to field applications requires validation under actual use conditions, documented antifouling architectures and maintenance or regeneration strategies, and a clear definition of the role of biosensors as monitoring or screening tools that complement reference methods where regulatory documentation is required. This requirement is equally critical for biosensors targeting fermentation metabolites and phenolic compounds, as their practical value depends not only on sensitivity but also on their ability to maintain stable and interpretable responses within the chemically evolving wine matrix.

Regulatory compliance is as decisive as technological performance. Treating the tank and sensing configuration as a unified system of food-contact materials, the incomplete harmonization of specific measures for certain material categories (especially cementitious or concrete), and increased requirements for nanomaterials and functional barriers in active or intelligent systems require a “regulatory-by-design” approach. Overall, the review concludes that mature and safe use of biosensors in fermentation is feasible only through a combination of realistic selection of in situ or at-line approaches by analyte and use, validation in the real matrix with emphasis on long-term stability, and a coherent compliance strategy for the complete food-contact system. Importantly, this framework should include not only safety-related targets but also biosensor platforms for process control and quality monitoring, especially those addressing fermentation metabolites and phenolic evolution.

The field is now moving toward a transition from “proof of concept” to standardized evaluation and comparable documentation: unified validation protocols under real fermentation conditions are needed, focused on long-term stability, interference and fouling management, and reproducibility across varieties, matrices, and tank materials. In parallel, integrating sensors into architectures that facilitate maintenance, recalibration, and hygienic design, as well as early alignment with food-contact material requirements (including functional barriers and restrictions on nanomaterials), will determine whether proposed solutions become reliable tools for process surveillance and compliance in the wine industry. From this broader perspective, the future relevance of biosensors in winemaking will depend not only on their ability to detect contaminants or verify compliance, but also on their capacity to support dynamic process control and quality-oriented decision-making during fermentation.

## Figures and Tables

**Table 1:** From fermentation vessel materials to Critical Control Points (CCPs): migration-driven risks during alcoholic fermentation.

Fermentation Vessel Material	Main Migrants Reported	Dominant Migration Mechanisms	Relevant CCP During Fermentation	Key Food Safety Implications	Possible Indirect Process/Quality Indicators	Ref.
Stainless steel (304/316)	Ni^2+^, Cr^3+^, Fe^2+^/Fe^3+^	Acidic pH, ethanol, SO_2_, weakened passive layer, aggressive CIP procedures	Chemical migration from metallic surfaces during active fermentation	Potential allergenic response (Ni), metal accumulation; requires surface integrity monitoring and controlled cleaning practices	Redox shifts, acetaldehyde increase, phenolic oxidation, color instability	[17,19,22,23,89,94]
Ceramic vessels	Pb^2+^, Cd^2+^	Glaze composition and quality, aging, firing temperature, acidic ethanolic matrices	Heavy metal release from glazed or degraded ceramic surfaces	Toxicological risk; high variability in artisanal vessels; glaze stability is a critical safety determinant	Fermentation inhibition, altered ethanol/organic acid balance, possible phenolic interactions	[28,30,95,96,97]
Concrete/cementitious tanks	Ca^2+^, K^+^, Al^3+^, trace metals	Hydration and carbonation reactions, Ca(OH)_2_ dissolution, porosity changes, interaction with acidic must	Alteration of ionic balance and pH during fermentation	Potential effects on yeast viability, tartrate stability, and regulatory uncertainty	Changes in fermentation kinetics, volatile acidity, ionic imbalance, altered mouthfeel and phenolic stability	[35,36,89]
Plastic containers (PET, HDPE, PP)	Sb, BPA, phthalates (DEHP, DBP), monomers/oligomers	Polymer composition, ethanol content, temperature, contact time, repeated use, material aging	Migration of organic additives during fermentation or prolonged contact	Risk of endocrine-disrupting compounds; increased risk with non-certified or degraded polymers	Mainly compliance-oriented; indirect process effects less specific, but possible quality deviations under prolonged contact	[41,44,98,99,100]

**Table 2:** Biorecognition elements used for selective detection and speciation of heavy metal ions.

Target Heavy Metal	Proposed Bioreceptor	Recognition Mechanism	Advantages	Limitations	Analytical Suitability	Ref.
Pb^2+^ (Lead)	DNAzymes	Pb^2+^-induced site-specific catalytic DNA cleavage	High selectivity under optimized ionic conditions; reversible response; chemical robustness	Performance dependent on controlled ionic environment	High	[108,109,110,121]
Cd^2+^ (Cadmium)	Aptamers	Target-induced structural reconfiguration	Low detection limits (pM–nM range); operational stability in ethanol-containing media	Susceptibility to nuclease degradation; chemical modification often required	High	[111,112,122,123]
Ni^2+^ (Nickel)	Molecularly imprinted polymers (MIPs)/Modified peptides	Chelation-based binding or synthetic cavity imprinting	Acidic pH tolerance; structural robustness; cost-effective fabrication	Generally lower binding affinity compared to highly specific nucleic acid receptors	Moderate–High	[114,124,125,126]
Cr(III)/Cr(VI)	DNAzymes/MIPs	Selective recognition enabling Cr(III)/Cr(VI) speciation	Capability for valence discrimination; suitability for targeted speciation	Increased sensor design complexity and calibration requirements	Moderate–High	[118,127,128]

**Table 3:** Transduction mechanisms in heavy metal sensing: Performance and limitations.

Transducer Type	Method	Main Targets	Advantages	Limitations	Suitability in Wine Matrices	Ref.
Electrochemical	ASV (anodic stripping voltammetry)	Pb^2+^, Cd^2+^, Zn^2+^	Very low LOD (ppb) enabled by preconcentration; favorable cost-to-sensitivity ratio	Susceptible to organic fouling; typically requires SPEs and surface modification	High (especially with SPEs and nanomodified surfaces)	[131,132,149]
Electrochemical	EIS/conductometric approaches	Ni^2+^, Cr(VI) (case-dependent)	Label-free readout; sensitive to interfacial changes	Complex signal interpretation; affected by ionic strength and matrix composition; requires strict control of conditions	Moderate (matrix-dependent)	[23,24]
Optical	Fluorescence (FRET/fluorescent reporters)	Al^3+^, Ni^2+^ (and others with suitable receptors)	High selectivity achievable via aptamers/DNAzymes; direct optical readout	Inner filter effect in red wine; photobleaching and matrix interference	High under optimized conditions (spectral window selection)	[113,141,142]
Optical	NIR platforms (UCNPs/NIR-emitting nanoprobes, QDs)	Al^3+^, Ni^2+^	Reduced color-related interference; improved S/N in complex matrices	Requires robust nanomaterial functionalization and stability; potential reproducibility constraints	High (particularly for red wine matrices)	[143,144,150]
Optical	Colorimetry (AuNPs)	Screening (multiple analytes)	Simple, equipment-free visual readout	Lower sensitivity; limited readability in red wines; often semi-quantitative	Limited (mainly for white/light-colored wines)	[14,104,151]
Mass-sensitive	QCM	Selected targets (e.g., Cr with appropriate receptors)	Label-free, real-time interfacial monitoring	Strongly affected by viscosity/density; requires stringent thermal/rheological control	Limited (primarily laboratory use)	[135,146]

**Table 4:** Analytical performance characteristics of biosensors versus reference methods for heavy metal analysis.

Performance Parameter	Biosensors	ICP-MS	GFAAS/ETAAS	Remarks in Fermentation Context	Ref.
Regulatory compliance (MRLs)	Suitable for routine screening/compliance when properly calibrated and matrix-matched	Reference method; fully compliant	Reference method; fully compliant	Key requirement is matrix-matched calibration and interference control in wine	[13,101]
Limit of detection (LOD)	Typically sub-μg/L to low-μg/L for electrochemical platforms (platform-dependent)	Very low LODs (trace/ultratrace)	Very low LODs (method- and matrix-dependent)	ICP-MS often exceeds routine needs; biosensor LODs can be sufficient if below regulatory thresholds	[21,131,132,160]
Sensitivity	Adequate for compliance-focused monitoring; boosted by nanomaterials and optimized transduction	Very high	Very high	“Fit-for-purpose” sensitivity is often more relevant than ppt-level capability in production monitoring	[23,104]
Selectivity	High with aptamers/DNAzymes/MIPs; matrix-dependent	High (targeted quantification; speciation with coupling)	High (with optimized protocols/modifiers)	Wine polyphenols and organic acids can affect sensor response; selectivity depends on receptor and surface	[103,109,110]
Matrix effect tolerance	Moderate–high (depends on antifouling design, surface chemistry, and receptor)	High with appropriate preparation/controls	High with appropriate preparation/controls	Polyphenols can cause fouling; surface engineering/cleanup improves robustness	[101,135,161]
Recovery (spiked wine)	Generally within acceptable ranges when tested in authentic wine matrices (method-dependent)	Typically high with validated protocols	Typically high with validated protocols	Spiking in real wine (not only buffer) is critical for validation relevance	[13,101]
Precision (RSD%)	Often <10% in optimized systems	Superior precision	High precision	Reference methods generally provide better repeatability; biosensors can be sufficient for process control	[13,101]
Sample preparation	Minimal to simplified protocols possible (often dilution/targeted cleanup)	Usually requires digestion/protocols for robust quantification	Usually requires digestion/protocols for robust quantification	Major operational advantage for in-process fermentation monitoring	[8,101,161]
Analysis time	Minutes	Hours including preparation	Hours including preparation	Key advantage for rapid screening during fermentation or quality control	[23,104]
Operational stability/reuse	Platform-dependent; nucleic acids and MIPs often more robust than enzymes in harsh matrices	Instrument-based stability	Instrument-based stability	Ethanol and low pH challenge enzyme-based sensors; receptor choice affects stability	[103,104]
Cost per analysis	Low–moderate	High	Moderate–high	Biosensors can reduce cost for high-throughput screening	[23,24]
Field deployability	Possible (portable/disposable formats)	Laboratory-based	Laboratory-based	Enables on-site monitoring in fermentation/production environments	[23,104]

**Table 5:** Performance and suitability of different biorecognition elements for ion sensing in complex winemaking environments.

Target Ion(s)	Biorecognition Strategy	Key Advantages	Main Limitations	Suitability in Fermentation Matrix	Ref.
Ca^2+^, Mg^2+^, K^+^	Molecularly Imprinted Polymers (MIPs)	High chemical stability; resistance to low pH and ethanol; structural robustness	Template leakage (in some systems); synthesis complexity	High suitability for long-term monitoring	[164,165]
Ca^2+^, Mg^2+^, K^+^	Ionophores (Potentiometric)	Fast response; low energy consumption	Cross-sensitivity among alkaline earth ions	Moderate; improved when used in sensor arrays	[169,170,171]
Fe^2+^/Fe^3+^	Siderophores (e.g., pyoverdine)	High affinity binding	Reduced selectivity in complex matrices; competitive binding	Moderate; matrix-dependent	[5]
Cu^2+^	Functionalized heterocyclic ligands	Strong chelate formation; nanomolar LOD; acid tolerance	Surface fouling risk	High suitability with proper surface engineering	[5,173,174,175]
Zn^2+^	Aptamers	Regeneration capability; structural resilience; high selectivity	Possible conformational changes in high ethanol	High suitability for continuous monitoring	[163,176]

**Table 6:** Signal transduction mechanisms for ion sensing and their suitability for fermentation monitoring.

Transduction Type	Typical Applications	Key Advantages	Main Limitations in Fermentation Matrix	Suitability for Continuous Monitoring	Ref.
Potentiometric (ISE, FET)	Ca^2+^, Mg^2+^, K^+^	Fast response; low energy consumption; minimal sample preparation	Baseline drift; surface fouling by polyphenols/proteins	High, with surface modification	[134,169,177]
Conductometric	Ionic strength monitoring; macroion trends	Simple configuration; real-time measurement	Limited selectivity; matrix conductivity variability	Moderate	[5,134,162]
Voltammetric (when applicable)	Selected redox-active ions	Good sensitivity; adaptable platforms	Fouling; requires stable reference electrode	Moderate to high	[5,172,178,179]
Optical (Fluorescence, SPR)	Selective ion detection	High selectivity; adaptable reporter systems	Inner filter effects; matrix coloration interference	Moderate	[5,181,184,185]
Near-Infrared/Nanophotonic Systems	Reduced matrix interference detection	Improved optical transparency window; enhanced sensitivity	Instrumental complexity; cost	Moderate	[5,183]
Quartz Crystal Microbalance (QCM)	Label-free binding studies	High sensitivity; direct mass detection	Sensitive to viscosity and temperature fluctuations	Low to moderate in industrial settings	[5,134,135]

**Table 7:** Analytical performance characteristics of biosensors versus reference methods for ions analysis.

Performance Parameter	Biosensors	ICP-MS/AAS	Remarks in Fermentation Context	Ref.
Regulatory Compliance (SMLs)	Generally within required SML ranges when properly calibrated	Fully compliant	Biosensors support routine compliance screening and process alarms; ICP-MS/AAS used for confirmation when needed.	[60,101,134]
Limit of Detection (LOD)	Typically nM range (platform-dependent)	Lower (often sub-µg/L; method-dependent)	ICP-MS/AAS achieve lower absolute LODs; biosensor LODs can still be sufficient for operational control and many regulatory thresholds.	[5,163,172]
Sensitivity	Adequate for routine control (“fit-for-purpose”)	Very high	Reference methods often exceed routine control needs; biosensors target actionable concentration ranges for timely process decisions.	[5,134,163]
Selectivity	High with MIPs/aptamers; matrix-dependent	Very high	Arrays + chemometrics mitigate cross-sensitivity (e.g., Ca^2+^/Mg^2+^) and improve selectivity under matrix variability during fermentation.	[168,170,186]
Matrix Effect Tolerance	Moderate → high (depends on surface/recognition element)	High (after digestion/ionization)	Polyphenols/ethanol and high ionic strength can drive fouling and signal drift; surface engineering and robust receptors are key for stable readings.	[5,180,182]
Recovery (Spiked Samples)	Typically ~90–110% (method-dependent)	Typically high	Spiking in authentic wines is essential to quantify matrix bias	[5,134]
Precision (RSD%)	Often <10% in optimized systems	Typically <2–3%	Reference methods retain superior precision; biosensors depend on baseline stability, fouling control, and calibration.	[5,134]
Sample Preparation	Minimal/simplified possible	Required (dilution/digestion/calibration)	Major operational difference: reference methods require digestion/dilution/calibration workflows; biosensors can run with minimal prep for near-real-time monitoring.	[5,134]
Analysis Time	Minutes/real-time	Longer (prep + instrument time)	Key advantage for kinetic monitoring and rapid corrective actions during fermentation, where delays reduce the value of the measurement.	[134,162]
Operational Stability	Platform-dependent; higher for MIPs/aptamers than enzymes	Instrument-based, high stability	Enzymes are more sensitive to ethanol/low pH; synthetic receptors (MIPs) and aptamers generally offer improved robustness for extended monitoring.	[134,180,182]
Cost per Analysis	Low → moderate	High	Biosensors advantageous for high-throughput screening; reference methods best reserved for confirmation and periodic audits.	[5,162,172]
Field Deployability	Possible (portable)	Laboratory-based	Enables on-site fermentation control and faster CAPA actions; lab confirmation used selectively near/exceeding thresholds.	[5,134,162]

**Table 8:** Comparison of biorecognition strategies for the detection of BPA and phthalates.

Target Compound(s)	Biorecognition Strategy	Key Advantages	Main Limitations	Suitability in Fermentation Matrix	Ref.
BPA	Enzymatic	High catalytic activity; simple integration; low material cost	Low substrate specificity; oxidation of natural polyphenols; ethanol-induced denaturation	Limited suitability due to cross-reactivity and reduced stability in acidic/ethanolic wine	[194,195,196]
BPA	Aptamers (DNA/RNA)	High molecular selectivity; reversible binding; regeneration capability	Sensitivity to ionic strength and ethanol concentration; conformational instability under harsh conditions	High suitability when properly optimized; compatible with real-time sensing	[197,198,199]
BPA	MIPs	High chemical and mechanical stability; resistance to low pH and ethanol; low production cost	Possible template leakage; batch-to-batch variability; synthesis complexity	Very high suitability for robust and disposable sensor platforms	[200,201,202]
PAEs	Aptamers	Target-specific recognition; adaptable to optical and electrochemical formats	Limited availability for certain phthalates; matrix interference possible	High suitability with optimized surface functionalization	[191,199]
PAEs	MIPs	Structural complementarity; strong selectivity; chemical robustness	Template bleeding; potential nonspecific adsorption	High suitability for long-term or industrial monitoring	[191,202]
BPA/PAEs	Antibodies	High affinity binding; well-established assay formats	Limited stability in ethanol; higher production cost; storage sensitivity	Moderate suitability; better for laboratory confirmation than continuous monitoring	[188]

**Table 9:** Signal transduction mechanisms for BPA and phthalates sensing and their suitability for fermentation monitoring.

Transduction Strategy	Nanomaterial Integration	Key Advantages	Main Limitations	Suitability in Fermentation Matrix	Ref.
Electrochemical (Glassy Carbon)	None or minimal	Simple setup; direct electrooxidation of phenolic groups	Limited sensitivity; slower electron transfer; surface fouling	Moderate suitability; improved with surface modification	[205,206]
Electrochemical (CNT- or GO-modified electrodes)	Carbon nanotubes (CNTs), Graphene oxide (GO)	Enhanced surface area; improved conductivity; higher S/N ratio	Potential fouling; fabrication variability	High suitability for portable and field-deployable systems	[209,215]
Composite Electrodes (Sonogel-Carbon)	Embedded nanomaterials in composite matrix	Mechanical robustness; improved reproducibility	More complex fabrication	High suitability for field monitoring	[210]
SPR (Surface Plasmon Resonance)	MIPs or aptamers on plasmonic surface	Real-time, label-free detection; high specificity	Instrumental complexity; higher cost	High analytical suitability; mainly laboratory-based	[199,211]
Fluorescence (FRET, Quantum Dots)	Quantum dots; nanostructured fluorophores	Very low LOD; strong signal amplification	Inner filter effect in red wines; optical interference	Moderate to high suitability with matrix correction strategies	[191,212,214]
Screen-Printed Electrodes (SPEs)	Carbon nanomaterials; metallic nanoparticles	Low cost; disposable; portable	Limited lifetime; calibration required	High suitability for industrial screening and on-site control	[205]

**Table 10:** Comparative overview of transducer types and platforms for BPA and phthalate monitoring.

Performance Parameter	Biosensors	GC-MS	HPLC-MS	Remarks in Fermentation Context	Ref.
Regulatory Compliance (SMLs)	Generally within required SML ranges when properly calibrated	Fully compliant	Fully compliant	Biosensors suitable for routine compliance screening	[188,216,219]
Limit of Detection (LOD)	Typically nM range (depending on platform)	Low ng/L or lower	Low ng/L or lower	Chromatographic methods provide lower absolute LODs	[208,212,218]
Sensitivity	Adequate for SML verification	Very high	Very high	Biosensors considered fit-for-purpose for industrial monitoring	[188,200,205]
Selectivity	High with MIPs/aptamers; matrix-dependent	Very high (after separation)	Very high (after separation)	Chromatography benefits from prior separation step	[199,220,221]
Matrix Effect Tolerance	Moderate to high (depends on recognition element and surface design)	High (after extraction/cleanup)	High (after extraction/cleanup)	Wine polyphenols may affect enzyme-based sensors	[191,195,205]
Recovery (Spiked Samples)	Typically 90–110%	95–105%	95–105%	Values consistent with accepted analytical performance criteria	[191,218,222]
Precision (RSD%)	Usually <10% in optimized systems	<2–3%	<2–3%	Reference methods retain superior precision	[191,217,221]
Sample Preparation	Minimal or simplified protocols possible	Required (extraction, SPE, cleanup)	Required (extraction, SPE, cleanup)	Major operational difference	[218,223,224]
Analysis Time	Minutes	Hours (including prep)	Hours (including prep)	Key advantage for screening applications	[216,222,225]
Operational Stability	Platform-dependent; higher for MIPs than enzymes	Instrument-based, high stability	Instrument-based, high stability	Enzymes more sensitive to ethanol and low pH	[199,200,208]
Cost per Analysis	Low to moderate	High	High	Biosensors advantageous for high-throughput screening	[205,216,217]
Field Deployability	Possible (portable formats)	Laboratory-based	Laboratory-based	Enables on-site fermentation control	[216,226,227]

**Table 11:** Comparison of biorecognition strategies used in biosensors for detecting major fermentation metabolites and phenolic compounds.

Target Analyte	Biorecognition Strategy	Key Advantages	Main Limitations	Suitability in Fermentation Matrix	Ref.
Ethanol	Alcohol dehydrogenase/Alcohol oxidase	High substrate specificity; strong compatibility with electrochemical and optical transducers; widely established biosensing strategy	Enzyme stability may decrease at high ethanol concentrations and under pH fluctuations	High suitability with appropriate immobilization strategies	[230,231,235]
Methanol	Alcohol oxidase/coupled enzymatic systems	Enables detection of low methanol concentrations; relatively simple enzymatic recognition	Limited selectivity versus ethanol and other primary alcohols	Moderate; improved using selective membranes or optimized configurations	[236]
Higher alcohols	Enzymatic approaches with limited substrate specificity	Potential for rapid screening in simplified matrices	Low molecular selectivity; difficulty distinguishing structurally similar alcohols; early stage of development	Low–moderate	[238]
Acetaldehyde	Aldehyde dehydrogenase	Good selectivity; suitable for electrochemical or optical signal conversion	Possible interference from other carbonyl compounds present in wine	Moderate–high with proper calibration	[54,239,241]
Malic acid	Malate dehydrogenase	High specificity; directly linked to monitoring malolactic fermentation	Enzyme stability dependent on matrix conditions	High	[247,250,259]
Lactic acid	Lactate dehydrogenase	Reliable monitoring of malolactic conversion; good enzymatic specificity	Possible interference from structurally related organic acids	High	[247,250,259]
Malic + Lactic acids	Bienzymatic systems	Simultaneous monitoring of key malolactic fermentation markers; improved process information	Increased sensor design and calibration complexity	High for targeted fermentation monitoring	[11,246]
Acetic acid	Whole-cell microbial biosensors	Reflects biological metabolic response; useful for detecting microbial spoilage	Lower analytical selectivity; biological signal interpretation can be complex	Moderate	[251]
Tartaric acid	Modified electrodes/conductive polymers	Structurally robust detection platforms; compatible with electrochemical sensing	Limited development of dedicated biorecognition elements; requires further validation in wine matrices	Moderate	[243,244,252]
Phenolic compounds	Phenol oxidases (laccase, tyrosinase)	Simple enzymatic recognition; compatible with electrochemical and optical platforms; suitable for total phenolic index estimation	Functional rather than molecular selectivity; response often reflects overall oxidizable phenolic pool	High suitability for screening and process monitoring	[253,254,260]

**Table 12:** Comparison of signal transduction strategies used in biosensors for the detection of fermentation metabolites and phenolic compounds.

Transduction Type	Typical Applications	Key Advantages	Main Limitations in Wine Matrix	Suitability in Fermentation Monitoring	Ref.
Electrochemical (amperometric/potentiometric/voltammetric)	Ethanol, acetaldehyde, organic acids, phenolic compounds	High sensitivity; compatibility with miniaturized and portable devices; relatively low cost; suitable for on-site applications	Electrode fouling; signal drift; matrix interference from polyphenols, pigments, and other electroactive species	High, especially with modified electrodes and antifouling strategies	[5,134,258,263,264]
Optical (absorbance/fluorescence/spectroscopic)	Fermentation metabolites; phenolic compounds; color- and oxidation-related parameters	High analytical sensitivity; no direct electrochemical contact with the sample; suitable for indirect enzymatic detection (e.g., NADH-based systems)	Strong light absorption and scattering in wine, especially in red wines; reduced signal-to-noise ratio; higher instrumental complexity and cost	Moderate; more suitable for controlled at-line or laboratory-supported applications	[240,266,273]
Conductometric	Selected metabolite-related measurements; reaction-induced conductivity changes	Simple sensing principle; potentially suitable for rapid measurements	Limited selectivity; strongly influenced by the intrinsic conductivity and complexity of wine matrix	Low–moderate	[5]
Hybrid electrochemical/optical platforms	Complex analytes requiring improved robustness or complementary readout	Combine sensitivity of electrochemical systems with broader analytical flexibility; potentially improved selectivity	Increased methodological complexity; more demanding optimization and calibration in real wine matrices	Moderate–high, depending on platform design	[148,274]

**Table 13:** Analytical performance characteristics of biosensors for key fermentation metabolites and phenolic compounds.

Performance Parameter	Biosensors	GC/HPLC	HPLC-UV/HPLC-DAD	UV-Vis/FT-MIR/FT-NIR	Remarks in Fermentation Context	Ref.
Regulatory Compliance	Suitable for routine process screening and rapid quality assessment when properly calibrated	Fully compliant for targeted laboratory analysis	Fully compliant for validated phenolic profiling and compositional analysis	Suitable mainly for screening, pattern recognition, and chemometric classification rather than confirmatory compliance	Biosensors support routine monitoring and rapid decision-making, whereas laboratory methods remain the reference tools for formal confirmation	[275,276,277]
Limit of Detection (LOD)	Typically in the μM to low mM range for metabolites; application-dependent for phenolic signals	Low, compound-specific	Low, especially for targeted phenolics	Usually higher than chromatographic methods; depends strongly on chemometric model and matrix	Chromatographic techniques generally provide lower absolute LODs, whereas biosensors and spectroscopic methods are often sufficient for process monitoring	[253,254,260,283,284]
Sensitivity	Adequate for fit-for-purpose monitoring	Very high	Very high	Moderate to high, depending on analyte class and calibration model	Biosensors and spectroscopic methods are generally fit-for-purpose for industrial monitoring, while chromatography is superior for detailed compositional analysis	[279,283]
Selectivity	Good in optimized systems, but often matrix- and target-dependent; frequently functional rather than molecular for phenolics	High after chromatographic separation	High after chromatographic separation; particularly suitable for phenolic classes	Lower intrinsic selectivity; relies heavily on spectral deconvolution and chemometric processing	Chromatography benefits from prior separation, while biosensors and spectroscopic tools may respond to broader analyte classes or matrix-dependent signatures	[253,256,277]
Matrix Effect Tolerance	Moderate to high, depending on recognition element, antifouling strategy, and calibration	High after sample preparation	High after extraction/dilution and chromatographic separation	Moderate; spectral overlap and matrix absorbance/scattering can be substantial, especially in red wines	Wine pigments, polyphenols, ethanol, and organic acids can strongly affect biosensor and spectroscopic responses if matrix correction is insufficient	[264,265,277,278,285]
Recovery (Spiked Samples)	Typically 85–110% in optimized systems	Typically high	Typically high	Model- and matrix-dependent; usually evaluated indirectly through prediction accuracy	Recovery remains a key validation criterion for biosensors and chromatography, while spectroscopic methods are assessed more often through calibration/prediction performance	[253,254,260,283,284]
Precision (RSD%)	Usually <10% in optimized systems	Generally superior	Generally superior	Dependent on calibration stability, spectral reproducibility, and preprocessing	Chromatographic methods retain better precision overall; biosensors and spectroscopic methods may be more affected by matrix variability and sensor/model drift	[253,254,260,283,284]
Sample Preparation	Minimal or simplified protocols possible	Usually required (filtration, dilution, extraction, derivatization depending on analyte)	Usually required (filtration, dilution, extraction)	Minimal to moderate; often limited to filtration/dilution, but strong dependence on calibration set quality	This is a major operational difference: biosensors and spectroscopic methods are generally faster and require less sample handling than laboratory chromatography	[279,280,281]
Analysis Time	Minutes	Longer, including sample preparation and run time	Longer, including sample preparation and run time	Rapid, often minutes once models are established	Biosensors and optical/spectroscopic tools offer clear advantages for rapid screening and process monitoring during fermentation	[5,7,281]
Operational Stability	Platform-dependent; limited by enzyme deactivation and/or electrode passivation	High instrumental stability	High instrumental stability	High instrumental stability, but prediction robustness depends on model maintenance and matrix consistency	In biosensors, ethanol, low pH, and fouling are critical; in spectroscopic systems, robustness depends more on calibration transfer and model drift than on sensor chemistry	[257,264,278]
Cost per Analysis	Low to moderate	Moderate to high	Moderate to high	Low to moderate after initial instrument/model investment	Biosensors and spectroscopic systems are advantageous for frequent screening, whereas chromatography is more costly but analytically stronger for confirmatory work	[279,280]
Field Deployability	Possible (portable/at-line/on-site formats)	Laboratory-based	Laboratory-based	Moderate to high, especially for portable/at-line optical systems	Biosensors and selected optical platforms enable near-line winery monitoring of fermentation progress and phenolic evolution, whereas chromatography remains laboratory-centered	[7,263,280,281]

**Table 14:** Application of biosensor platforms for CCP monitoring and HACCP compliance across different fermentation vessel types.

Fermentation Vessel Type	Associated CCP	Primary Monitoring Target	Proposed Biosensor Platform	HACCP Function	Corrective Action Trigger	Ref.
Stainless Steel Tanks	Metal ion release due to corrosion	Ni^2+^, Cr^3+^/Cr^6+^, Fe species	MIP-based or aptamer-based metal sensors	CCP Monitoring	Exceedance of internal alert limits → inspection, passivation, or tank replacement	[133,292,295]
Ceramic Vessels	Heavy metal migration from glaze	Pb^2+^, Cd^2+^	DNAzyme- or aptamer-based electrochemical sensors	CCP Monitoring	Upward concentration trend → suspension of vessel use	[108,110,291]
Concrete/Cementitious Tanks	Ionic imbalance and potential trace element leaching	Ca^2+^, K^+^, Al^3+^, trace elements	Ion-selective electrochemical biosensors	CCP and Process Monitoring	Ionic deviation → adjustment of process parameters or vessel evaluation	[292,293]
Plastic Fermentation Vessels	Migration of organic additives	BPA, phthalates, residual monomers	MIP-based or nano-enhanced electrochemical biosensors	CCP Monitoring	Detection above threshold → material withdrawal or replacement	[188,200]
All vessel types	Indirect process deviation linked to vessel–matrix interaction	Ethanol, organic acids, acetaldehyde, phenolic profile/redox-related phenolic changes	Enzymatic and electrochemical biosensors for fermentation metabolites and phenolics	Supportive process monitoring	Deviation from expected fermentation or phenolic evolution pattern → targeted inspection and confirmatory analysis	

**Table 15:** Proposed deployment strategies and decision-making frameworks for biosensor-based monitoring of critical analytes in wine fermentation.

Analyte Category	Associated Control Point (CCP/Process Control)	Recommended Deployment Strategy	Measurement Frequency	Decision Type
K^+^, Ca^2+^, Mg^2+^	Process Control—Ionic balance and stabilization	In situ (Ion-Selective Electrodes)	Continuous	Real-time process adjustment (cooling, stabilization control)
Fe^2+^/Fe^3+^, Cu^2+^	Process Control—Redox management	In situ or At-line (speciation-capable platforms)	Periodic or semi-continuous	Oxygenation control/redox correction
Ethanol, organic acids, acetaldehyde	Process Control—Fermentation kinetics/malolactic progression/oxidative status	In situ, bypass-flow, or At-line biosensor platforms	Continuous, semi-continuous, or checkpoint-based	Fermentation adjustment; detection of sluggish/stuck fermentation; malolactic control; corrective process intervention
Phenolic compounds	Process/Quality Control—Extraction management, color evolution, oxidation control	At-line/On-site; selected in-line optical systems where available	Periodic or high-frequency checkpoint monitoring	Maceration control; pressing timing; oxidation management; aging-related quality adjustment
Pb^2+^, Cd^2+^, As	CCP—Chemical safety	At-line (Disposable SPEs with ASV)	Batch-based	Accept/reject decision; regulatory compliance screening
Bisphenol A, Phthalates	CCP—Migration from vessel materials	At-line (MIP- or aptamer-based platforms)	Batch release	Compliance verification (SML-based pass/fail)

## Data Availability

This review is based exclusively on previously published data and peer-reviewed literature, all of which are cited within the manuscript.

## Abbreviations

The following abbreviations are used in this manuscript:

AAS	Atomic Absorption Spectrometry
AOAC	Association of Official Analytical Chemists
ASV	Anodic Stripping Voltammetry
AuNPs	Gold Nanoparticles
BPA	Bisphenol A
CaCO_3_	Calcium Carbonate
Ca(OH)_2_	Calcium Hydroxide
CCP	Critical Control Point
CIP	Cleaning-In-Place
CNTs	Carbon Nanotubes
DBP	Dibutyl Phthalate
DEHP	Di(2-ethylhexyl) Phthalate
DMG	Dimethylglyoxime
EIS	Electrochemical Impedance Spectroscopy
ETAAS	Electrothermal Atomic Absorption Spectrometry
FCM(s)	Food Contact Material(s)
FET	Field-Effect Transistor
FRET	Fluorescence Resonance Energy Transfer
GC-MS	Gas Chromatography–Mass Spectrometry
GFAAS	Graphite Furnace Atomic Absorption Spectrometry
GO	Graphene Oxide
HACCP	Hazard Analysis and Critical Control Point
HDPE	High-Density Polyethylene
HPLC-MS	High-Performance Liquid Chromatography–Mass Spectrometry
ICP-MS	Inductively Coupled Plasma–Mass Spectrometry
ISE	Ion-Selective Electrode
ISO	International Organization for Standardization
Kaff	Affinity Constant
KHT	Potassium Bitartrate
kon/koff	Association/Dissociation Rate Constants
LOD(s)	Limit(s) of Detection
MIP(s)	Molecularly Imprinted Polymer(s)
MRL(s)	Maximum Residue Limit(s)
NIR	Near-Infrared
OIV	International Organisation of Vine and Wine
OML	Overall Migration Limit
PAE(s)	Phthalate Ester(s)
PAT	Process Analytical Technology
PET	Poly(ethylene terephthalate)
PP	Polypropylene
QCM	Quartz Crystal Microbalance
QDs	Quantum Dots
Rct	Charge Transfer Resistance
RSD	Relative Standard Deviation
SNR	Signal-to-Noise Ratio
SML(s)	Specific Migration Limit(s)
SME(s)	Small and Medium-sized Enterprise(s)
SO_2_	Sulfur Dioxide
SPE(s)	Screen-Printed Electrode(s)
SPR	Surface Plasmon Resonance
Tsat	Saturation Temperature
UCNPs	Upconversion Nanoparticles
